# Immunocompatible Elastomer With Enhanced Fibrous Capsule‐Resistance and Elasticity by Water‐Induced Surface Phase Reconfiguration.

**DOI:** 10.1002/advs.202504801

**Published:** 2025-06-25

**Authors:** Xianchi Zhou, Wenzhong Cao, Weifeng Liu, Wenbin Dai, Zihao Zhu, Fan Jia, Haijie Han, Ke Yao, Youxiang Wang, Jian Ji, Peng Zhang

**Affiliations:** ^1^ State Key Laboratory of Transvascular Implantation Devices The Second Affiliated Hospital School of Medicine Zhejiang University Hangzhou 311202 P. R. China; ^2^ MOE Key Laboratory of Macromolecule Synthesis and Functionalization of Ministry of Education Department of Polymer Science and Engineering Zhejiang University Hangzhou 310058 P. R. China; ^3^ Department of Hepatobiliary and Pancreatic Surgery The Second Affiliated Hospital School of Medicine Zhejiang University Hangzhou 310009 P. R. China; ^4^ Key Laboratory of Cardiovascular Intervention and Regenerative Medicine of Zhejiang Province Department of Cardiology Sir Run Run Shaw Hospital School of Medicine Zhejiang University Hangzhou 310016 P. R. China; ^5^ Eye Center The Second Affiliated Hospital School of Medicine Zhejiang University Zhejiang Provincial Key Laboratory of Ophthalmology Zhejiang Provincial Clinical Research Center for Eye Diseases Zhejiang Provincial Engineering Institute on Eye Diseases Hangzhou Zhejiang 310009 China; ^6^ Transvascular Implantation Devices Research Institute P. R. China Hangzhou 310009 China

**Keywords:** foreign body response, immunocompatible elastomer, implant, phase reconfiguration, surface‐bulk heterogeneity

## Abstract

Integrating surface and bulk heterogeneity enables synergistic optimization of implantable biomaterials, harmonizing surface‐tissue interactions with the mechanical integrity of the bulk. However, achieving this heterogeneity within a single material remains a significant challenge. Here, a strategy to engineer spontaneously generated heterogeneous surface and bulk structures within a material is introduced. The resulting material is termed phase‐separation and underwater reconfiguration‐enhanced (PURE) elastomers. PURE elastomers integrate the high elasticity of the bulk with superior immunocompatibility at the surface. By de novo designing the immunomodulatory and alkyl acrylate monomer structures, this is regulated that the copolymer's spatial configurations, ultimately influencing its condensed structure. Tuning the alkyl side chain length facilitates the formation of a distinctive phase‐separation pattern in elastomers that accommodates large deformations, resulting in excellent toughness and low hysteresis. Moreover, this phase structure supports a water‐induced surface phase reconfiguration, aligning with in vivo applications. This reconfiguration enriches immunomodulatory groups at the material surface, significantly enhancing immunocompatibility. Consequently, these elastomers exhibit extremely low fibrotic capsule formation for up to one year in mice and two months in non‐human primates. This findings introduce a class of durable, fibrosis‐resistant materials and establish a new strategy for designing heterogeneous polymeric elastomers with broad biomedical applicability.

## Graphical abstract

This study introduces a strategy for engineering heterogeneous surface and bulk structures within a single material, termed phase‐separation and underwater reconfiguration‐enhanced (PURE) elastomers. These elastomers combine high bulk elasticity with enhanced immunocompatibility at the surface. By tuning alkyl side chain lengths, PURE elastomers support water‐driven surface phase reconfiguration, improving immunomodulatory properties and minimizing fibrotic response in vivo.

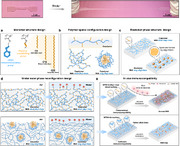

## 1 Introduction

Structural heterogeneity between the surface and bulk is highly desired for implantable biomaterials and devices.^[^ ^1^ ^]^ Upon implantation, the biomaterial's surface is the frontline interface for interactions with surrounding tissues.^[^ ^2^, ^3^ ^]^ The interplay between proteins, cells, and the material surface is critical in determining its functionality.^[^ ^4^, ^5^, ^6^, ^7^ ^]^ Surface functionalization is pivotal in regulating critical biological processes, including coagulation,^[^ ^8^, ^9^, ^10^ ^]^ bacterial adhesion,^[^ ^11^ ^]^ tissue regeneration,^[^ ^12^ ^]^ friction,^[^ ^13^ ^]^ immune response,^[^ ^14^ ^]^ and other complications. However, for the bulk of the material, the emphasis is on ensuring sufficient mechanical properties to withstand prolonged implantation in vivo.^[^ ^15^ ^]^ Therefore, the structural heterogeneity between the surface and bulk is essential to fulfill distinct functional requirements. Despite its importance, realizing this heterogeneity within a single material remains a significant challenge.

The foreign body response (FBR) poses a significant challenge to implants.^[^ ^16^, ^17^, ^18^, ^19^, ^20^, ^21^, ^22^, ^23^, ^24^, ^25^, ^26^, ^27^, ^28^, ^29^, ^30^, ^31^ ^]^ Upon implantation, the material surface undergoes a dynamic and intricate interplay with a diverse repertoire of proteins and cell types, necessitating a sophisticated immunocompatibility design to ensure a favorable biological response.^[^ ^27^, ^32^, ^33^, ^34^, ^35^, ^36^, ^37^ ^]^ Meanwhile, long‐term implantation demands robust mechanical properties in the material bulk.^[^ ^38^, ^39^ ^]^ As such, surface and bulk heterogeneity have emerged as one of the critical design principles for developing anti‐FBR materials, with the ideal goal being to achieve stable intrinsic heterogeneity within a single material to coordinate different functional requirements. Some studies have explored strategies to mitigate FBR within a single elastomeric material system. An early study introduced zwitterionic components into polyurethane to confer intrinsic hydrophilicity and mitigate the FBR.^[^ ^40^ ^]^ However, the design did not account for material heterogeneity, and the introduction of hydrophilic components would cause swelling, which would adversely affect its mechanical properties. Recently, we developed a coating‐free elastomer that achieves heterogeneity through in situ generation of anti‐FBR zwitterionic surfaces.^[^ ^41^ ^]^ Yet, this approach requires additional pre‐treatment before use.

**Scheme 1:**
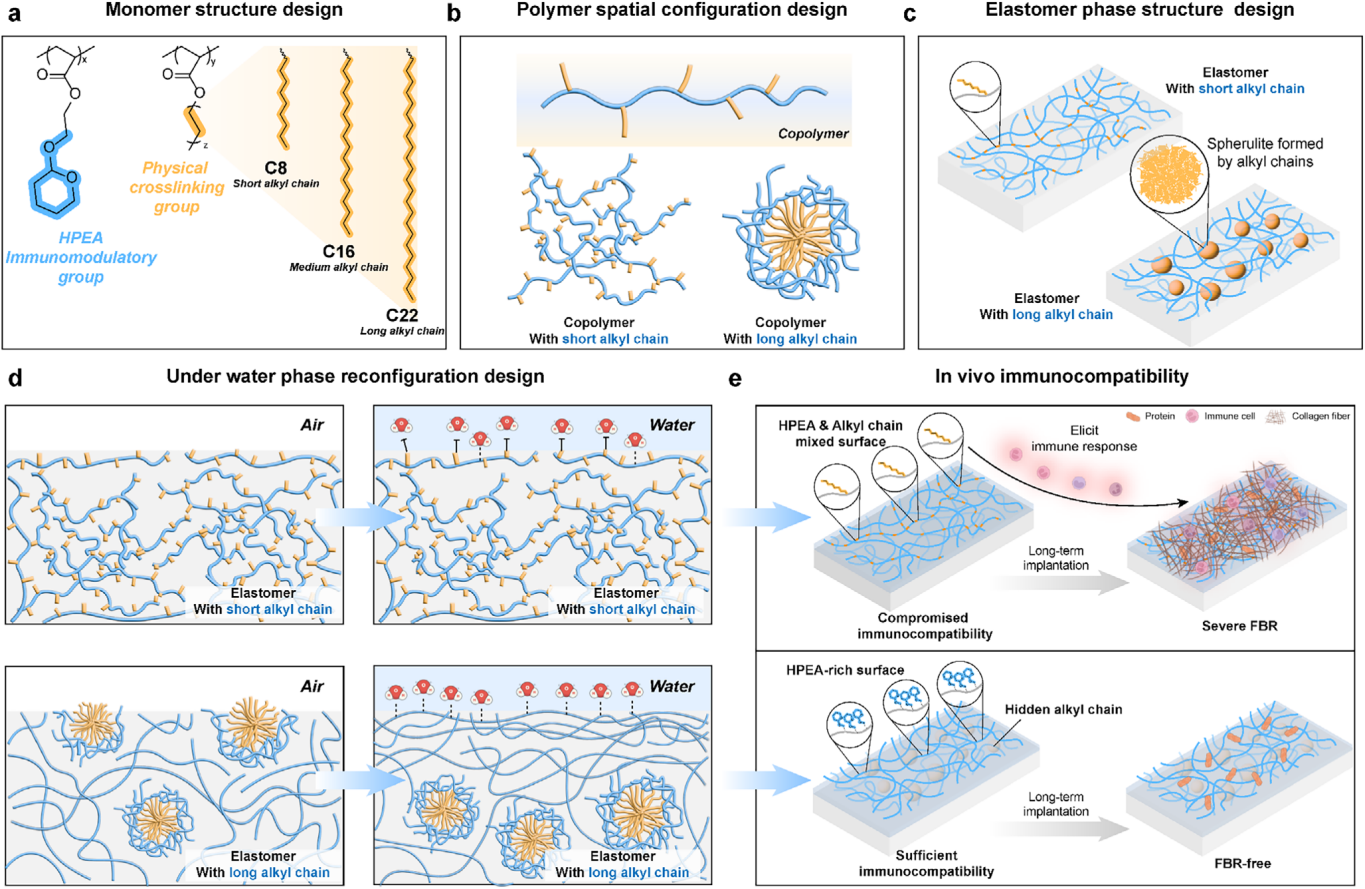
Schematics of the immunocompatibility design of PURE elastomers. a) The chemical structure of the monomers used for preparing PURE elastomers. b) Schematic diagram of the spatial configuration of polymers. c) Schematic diagram of the phase separation structure of PURE elastomers. d) Schematic diagram of water‐induced phase reconfiguration in PURE elastomers. e) Schematic diagram of FBR performance of PURE elastomers in vivo.

Our previous work found that monomers with a tetrahydropyran (THP) ring can work as immunoregulatory units to create immunocompatible hydrophobic elastomer systems (named EVADE) through the copolymerization of a small amount of octadecyl methacrylate to form aggregation domains for physical cross‐linking.^[^ ^42^ ^]^ However, the lack of structural heterogeneity in the design fails to achieve the preferential enrichment of THP groups on the elastomer surface, and the bulk elasticity of the elastomer remains significantly insufficient. In this work, we report a strategy for constructing an intrinsically heterogeneous surface and bulk structure within materials. The PURE elastomers prepared using this strategy exhibit spontaneously formed heterogeneous surface and bulk structures, combining high bulk elasticity with surface immunocompatibility, making them suitable for implantable biomaterials. To address limitations in the former study, we sought to design the monomer structure of the elastomer from the ground up, aiming to adjust the spatial configuration of the copolymer and further regulate the condensed state structure of the elastomer, thereby imparting structural heterogeneity. A previous study has shown that the side chain length of alkyl acrylates in binary copolymers influences the copolymer configuration in solution. Shorter side chains tend to form fluffy aggregates, while longer side chains favor the formation of hydrophobic domains.^[^ ^43^ ^]^ This insight inspired us to design a binary copolymer system comprising the immunoregulatory HPEA units and alkyl acrylates with diverse side chain lengths to prepare elastomers with different phase structures (**Scheme** **1**a). With this design, this spatial configuration adjustment of copolymers also applies to the elastomeric systems (Scheme 1b). By optimizing the side chain length, we obtain an elastomer formulation with a phase‐separated structure featuring micron‐scale crystalline domains rich in alkyl chains embedded within the elastomeric matrix enriched with THP groups (Scheme 1c). The presence of microcrystals serves as physical crosslinking points that effectively restrict molecular chain slippage, thereby reducing hysteresis and enhancing the elastomer's elasticity. Moreover, the aggregation of alkyl chains forms large crystal domains in the optimal elastomer, which, in turn, implies the presence of a large number of freely moving polymer chains rich in relatively hydrophilic THP groups, enabling phase reconfiguration upon water induction. (Scheme 1d). This unique water‐induced phase reconfiguration enables the enrichment of surface immunoregulatory units and thus imparts extraordinary immunocompatibility to the PURE elastomer (Scheme 1e).

## 2 Results

### 2.1 Water‐Induced Surface Phase Reconfiguration of PURE Elastomers

**Figure 1:**
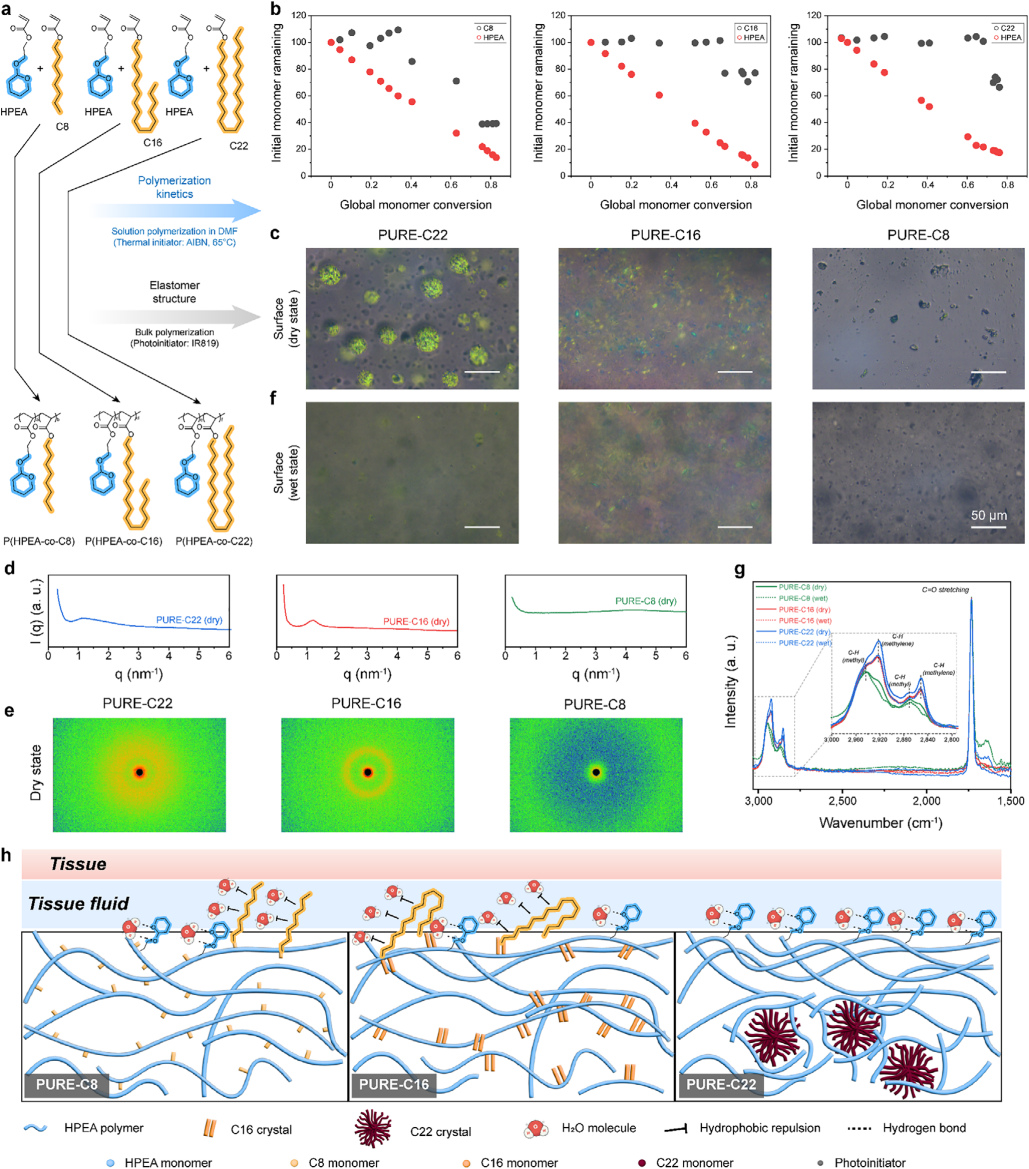
Polymerization kinetics and water‐induced phase reconfiguration of PURE elastomers. a) Schematic diagram of experimental design. The polymerization kinetics experiment was conducted through solution polymerization b). The elastomers were prepared by bulk polymerization and observed under a polarizing microscope c,f). b) Individual monomer conversion as a function of global conversion. c,f) Polarized optical images of PURE elastomers with different alkyl acrylates under dry (c) and wet (f) states. The film thickness is 1 mm, and the polarized mode is in the presence of a 530 nm tint plate at azimuth angles of −45 ° and 45°. d,e) SAXS pattern (d) and 2D‐SAXS image (e) of PURE elastomers under dry state. g) ATR‐FTIR spectroscopy of PURE elastomers under dry and wet environments. h) Proposed mechanisms of water‐induced phase reconfiguration to enhance immunocompatibility.

We first synthesized the immunomodulatory monomer HPEA (Figure S1; Figure S2, Supporting Information) and investigated the polymerization kinetics of HPEA with three alkyl acrylates with varying side chain lengths through solution polymerization. PURE elastomers were subsequently prepared via bulk polymerization and characterized for their phase structure (**Figure** **1**a; Figure S3, Supporting Information). The kinetic data reveal that HPEA polymerizes at the onset of the reaction, while the three alkyl acrylates—*n*‐octyl acrylate (C8), hexadecyl acrylate (C16), and docosyl acrylate (C22)—remain inert initially. As the reaction proceeds, C8 participates in polymerization when the overall conversion rate reaches 33.6%. In contrast, C16 and C22 only begin to participate when the overall conversion reaches 64.6% and 68.1%, respectively (Figure 1b). These differences are likely due to the steric hindrance effects of the varying side chain lengths of the alkyl acrylates. This variation in reactivity influences the distribution of monomer units along the polymer chain, leading to compositional inhomogeneity within the copolymers.

Next, we investigated the internal microstructure of PURE elastomers synthesized via bulk polymerization of HPEA and different alkyl acrylics at a molar ratio of 90%:10% (designated as PURE‐C8, PURE‐C16, and PURE‐C22). By observing the surface of the elastomer using a polarization microscope, we observed spherical crystal structures with diameters of 20–50 µm on the surface of PURE‐C22. In contrast, wedge‐shaped crystals with diameters of several micrometers were observed on the surface of PURE‐C16. No apparent crystallization was observed on the PURE‐C8 surface (Figure 1c). Small‐angle X‐ray scattering (SAXS) was employed to characterize the internal structure of various PURE elastomer formulations. SAXS intensity (I) was plotted as a function of the scattering vector (q) (Figure 1d,e). In the low‐q region (q < 0.7 nm⁻¹), the scattering profiles indicated the presence of phase‐separated structures larger than 280 nm in the dry state. Notably, PURE‐C16 and PURE‐C22 exhibited significantly stronger scattering signals within this range compared to PURE‐C8, indicating the presence of large‐scale crystalline structures, aligning with observations from polarized light microscopy. This variation in elastomer structure can be attributed to two factors. First, longer alkyl side chains are more likely to adopt ordered arrangements, facilitating the formation of crystalline structures. Second, the polymerization kinetics indicate that as the alkyl chain length increases, alkyl acrylates participate more readily in the later polymerization stages. This leads to the accumulation of alkyl chains on the polymer chains formed during the last stages, promoting interactions among many alkyl chains across an extended region. This explains why spherical crystals can form up to tens of micrometers in size.

Based on previous observations of polymerization kinetics and the phase structure of elastomers, it is evident that the alkyl acrylic units with long alkyl chains in PURE‐C22 elastomers aggregate into crystalline spherulites of tens of microns. On the other hand, the remaining polymer chains, which are rich in immunoregulatory THP groups, are minimally cross‐linked, thereby retaining the potential for large‐scale motion. To investigate whether the elastomers undergo phase structure changes in a wet environment, we immersed them in water for a period and observed their phase behavior. Notable structural changes were observed in the PURE‐C22 formulation under wet conditions. In the wet state, all the spherical crystals on the surface of PURE‐C22 disappeared, and it was faintly visible that the spherulites appeared to migrate into the interior of the elastomer. The PURE‐C16 surface showed a slight reduction in wedge‐shaped crystals, while the surface of PURE‐C8 exhibited no significant changes (Figure 1f).

After observing this distinctive water‐induced phase reconfiguration behavior of PURE‐C22, we hypothesized that the disappearance of large spherulites from the surface would cause an increase in the immunocompatible THP groups on the surface, thereby improving its immunocompatibility. This change in abundance was confirmed by Attenuated Total Reflection Fourier Transform Infrared spectroscopy (ATR‐FTIR). As shown in Figure 1g, three types of characteristic peaks were observed. The intensity of the ester (C═O) absorption peak at 1726 cm^−1^ reflects the relative content of all acrylate units, and the intensity in all samples was adjusted to be consistent to compare the relative content of long alkyl chains on the surface. Long alkyl chains exhibit two distinct signals: the methyl peak (2870 and 2945 cm^−1^) at the end of the alkyl chain and the methylene peak (2850 and 2925 cm^−1^) at the middle of the chain. By maintaining a constant intensity for the ester peak (C═O) across all samples, changes in the relative content of the long alkyl chain on the elastomer surface can be observed through variations in the intensity of the methylene peak. In the dry state, the methylene peak intensities varied among different samples, with PURE‐C22 exhibiting the highest signal, followed by PURE‐C16, and PURE‐C8 showing the lowest. These differences are attributed to the varying numbers of methylene groups in the alkyl acrylates. Upon exposure to a water environment, the intensity of the methylene peak for the long alkyl chains on the surface of PURE‐C22 decreased markedly, which aligns with the observed loss of spherulites from the surface. In contrast, the reduction in the methylene peak signals for PURE‐C16 was less pronounced. For PURE‐C8, the methylene signal was inherently weak, making it difficult to compare the differences between the dry and wet states.

The above investigations suggest that the copolymer compositions in a single reaction can be varied due to the different reactivity ratios of alkyl acrylates. The C22 monomer tends to react in the late stage of polymerization, forming alkyl‐rich copolymers (Figure S4, Supporting Information). The unique composition distribution enables the formation of large spherulites in PURE‐C22 elastomers. Upon exposure to water, the relatively hydrophilic, immunomodulatory THP group‐rich molecular chains migrate to the water‐elastomer interface. This water‐driven phase reconfiguration substantially enhances the prevalence of THP groups on the PURE‐C22 surface under physiological humidity conditions (Figure 1h). Such a phase reconfiguration, characterized by the reduction of long‐chain carbons on the surface, demonstrates the potential of PURE‐C22 as a highly immunocompatible material for biomedical applications.

### 2.2 The Alkyl Chain Length of Alkyl Acrylates Influences the Elasticity of PURE Elastomers

**Figure 2:**
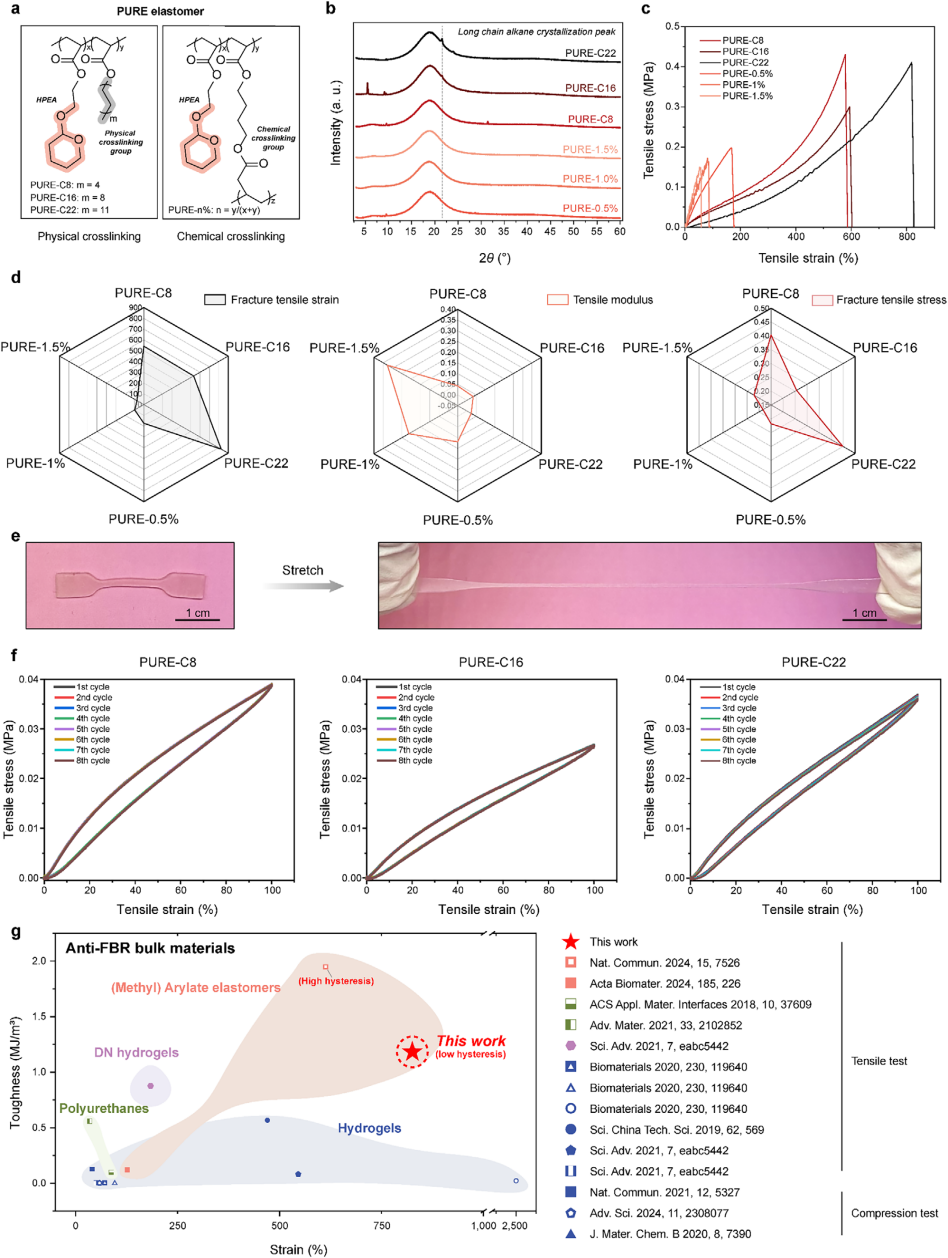
Characterization of PURE elastomers. a) PURE elastomers were prepared through chemical or physical crosslinking. For example, the elastomer prepared by adding a 0.5% mass fraction of chemical crosslinking agent is designated as PURE‐0.5%. b) XRD analysis of PURE elastomers. c) Stress‐strain curves of PURE elastomers at room temperature. Samples had a width of 2 mm, a thickness of 1 mm, and a gauge length of 12 mm, with a stretching speed of 10 mm min^−1^. d) Fracture tensile strain, tensile modulus, and fracture tensile stress of PURE elastomers. e) A tensile test sample of the PURE‐C22 elastomer was subjected to large deformation. f) Stress‐strain curves of PURE elastomers in cyclic tensile tests, with repeated stretching to 100% strain. Sample dimensions: 2 mm width, 1 mm thickness, and 12 mm gauge length, with a stretching speed of 10 mm min^−1^. g) Comparison of the toughness and fracture tensile/compression strain for PURE‐C22 and other anti‐FBR bulk materials recently reported in the literature.

To improve the elastomer elasticity, we synthesized an acrylate monomer, HPEA, instead of the methacrylate analog used in our previous study^[^ ^42^ ^]^ for higher polymer chain flexibility. The decrease in glass transition temperature (T_g_) from 23 °C for HPEMA homopolymer to −12 °C for HPEA homopolymer indicates improved polymer chain flexibility (Figure S5, Supporting Information). From the perspective of alkyl acrylates, we explored acrylic alkyl with varying side chain lengths to select the elastomer formulation with optimal mechanical properties. Additionally, we synthesized elastomers with chemical crosslinking for a comprehensive comparison (**Figure** **2**a; Table S1, Supporting Information).

Differential scanning calorimetry (DSC) testing indicated that all elastomers showed an apparent T_g_ lower than room temperature, confirming their rubbery state. Chemical crosslinking (CC) elastomers exhibited a T_g_ ≈0 °C, whereas physical crosslinking (PC) elastomers exhibited lower T_g_ values, ranging from −9 to −15 °C, indicating better polymer chain flexibility (Figure S6 and S7, Supporting Information). X‐ray diffraction (XRD) spectra demonstrated that the CC elastomers are amorphous, while the PC elastomer showed varied crystallization capabilities. PURE‐C22 exhibited a strong crystallization peak, while weak crystallization occurred for PURE‐C16, and PURE‐C8 did not crystallize (Figure 2b).

We further evaluated the mechanical properties of the elastomer through tensile tests (Figure 2c). Results show that PC elastomers exhibit significantly superior elasticity compared to CC counterparts. PC Elastomers exhibited fracture tensile strains exceeding 600% and fracture stress ranging from 0.25 to 0.4 MPa. However, the fracture tensile strain of CC elastomers is below 200%, and the fracture stress is less than 0.25 MPa (Figure 2d; Figure S8, and Table S2, Supporting Information). This improvement is attributed to the formation of localized ordered regions that serve as crosslinking domains within the amorphous matrix. These ordered regions contribute to a dynamic network structure that better accommodates stress variations, effectively reducing stress concentration.^[^ ^44^ ^]^ In contrast, due to their rigidity, CC networks tend to show diminished toughness under varying stress conditions. Among the PC elastomers, PURE‐C22, with the strongest crystallinity, demonstrated the highest elasticity (Figure 2e). Furthermore, the PURE‐C22 elastomer exhibits excellent rebound performance, as characterized by cyclic tensile tests. As shown in Figure 2f, the hysteresis of PURE‐C22 is represented by the calculated dissipation energy (2.83 kJ m^−3^) and the corresponding dissipation ratio (13.8%). This dissipation ratio is the lowest among the PC elastomers, with PURE‐C16 at 15.3% and PURE‐C8 at 17.1% (Table S3, Supporting Information). Figure 2g summarizes the toughness and fracture tensile/compression strain of the reported anti‐FBR bulk materials. PURE‐C22 elastomer showed significantly higher toughness and strain than these materials.^[^ ^15^, ^19^, ^38^, ^39^, ^40^, ^41^, ^42^, ^45^, ^46^, ^47^ ^]^ Although EVADE elastomers exhibit higher toughness, their severe hysteresis limits their applicability.

Next, rheological tests were conducted on all PURE elastomer samples to assess their behavior under small deformations. The relationship between storage modulus (*G′*) and loss modulus (*G″*) at 0.1 Hz is used to evaluate the rheological state of the samples.^[^ ^48^ ^]^ For all PURE elastomers, the *G'* was significantly higher than the *G“”* at 0.1 Hz across a wide frequency range, indicating a rubbery state (Figure S9, Supporting Information). The loss factor (tan δ) assesses damping performance. At a typical human motion frequency of 1 Hz, all PURE elastomers exhibited tan δ values below 0.4, indicating low energy loss and hysteresis (Figure S10, Supporting Information). Strain amplitude sweep curves revealed that all PURE elastomers, except for PURE‐C22, experienced significant non‐linear deformation (*G′* < *G″*) after ≈10% strain, whereas PURE‐C22 maintained elastic behavior (*G′* > *G″*) up to 100% strain (Figure S11, Supporting Information). When subjected to step‐strain changes, PURE‐C22 quickly returned to its original *G′* level after transitioning from high (100%) to low strain (1%), unlike PURE‐C8 and PURE‐C16, which showed poorer recovery (Figure S12, Supporting Information). This corresponds to the rapid recovery and low hysteresis of PURE‐C22 observed in the tensile test. Overall, PURE elastomers exhibit strong elasticity and low hysteresis, with PURE‐C22 demonstrating the best performance, making it suitable for implantable biomedical devices.

### 2.3 Evaluation of Inflammatory Response to PURE Elastomers

**Figure 3:**
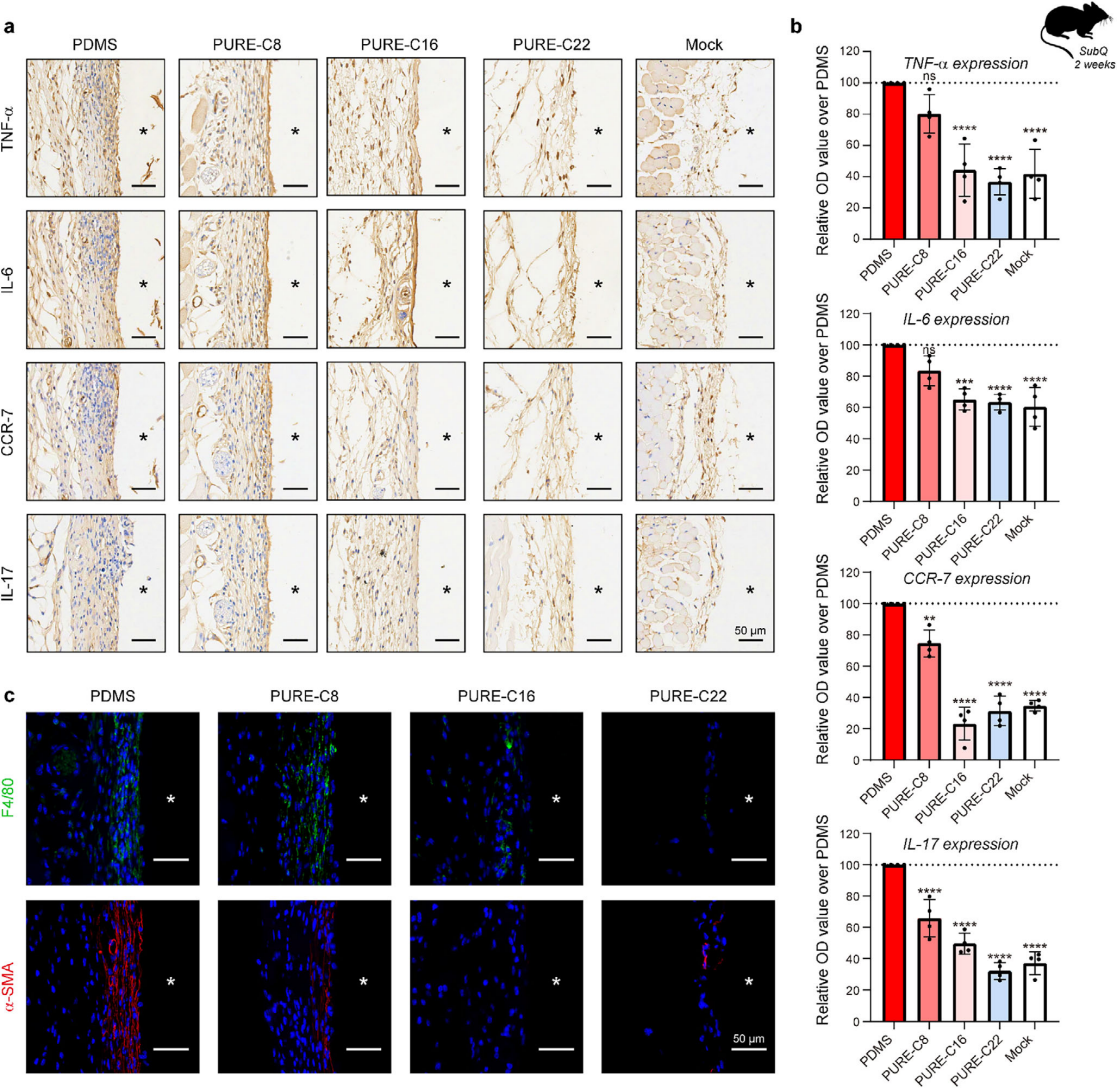
Evaluation of acute inflammatory responses induced by PURE elastomers. To assess the acute inflammatory response, subcutaneous (SubQ) implantation was performed using discs made of PDMS and PURE elastomers (PURE‐C8, PURE‐C16, and PURE‐C22), each with a diameter of 4 mm and a thickness of 1 mm. The surrounding tissue was analyzed 14 days post‐implantation to evaluate inflammation. In the histological sections, the asterisk “*” marks the original implant locations. a,b) Immunohistochemistry (IHC) was conducted to detect key inflammatory markers, including TNF‐α, IL‐6, CCR7, and IL‐17. Representative staining images are shown in a), while quantification is presented in b). Cells positive for inflammatory markers appear brown, whereas all nuclei are counterstained blue with hematoxylin. Data were collected from the tissue region within 50 µm of the tissue‐material interface (*n* = 3 mice per group, mean ± s.d.). c) Immunofluorescence staining of sections for F4/80 (green, macrophages) and α‐SMA (red, fibroblasts), and nuclei stained via DAPI (blue) (*n* = 3 mice per group). Statistical significance was determined by an unpaired, two‐tailed t‐test. **P < 0.01, ***P < 0.001, ****P < 0.0001, ns: not significant.

The excellent elasticity of PURE‐C22 ensures its potential for long‐term use in vivo. However, for implantable biomaterials, the ability to resist the formation of a fibrous capsule over time is crucial for maintaining their intended functionality. By designing the condensed state structure of PURE elastomers, we achieved water‐driven surface phase reconfiguration, which increases the prevalence of immunomodulatory THP groups on the surface. This highlights its potential as a highly immunocompatible material for biomedical applications. To validate our conceptual design, we assessed the in vivo inflammatory and fibrotic responses following the implantation of different PURE elastomers (PURE‐C8, PURE‐C16, and PURE‐C22). One of the most widely used medical elastomers, poly(dimethylsiloxane) (PDMS), was selected as a benchmark for implantation studies. It is well established that factors such as modulus,^[^ ^49^ ^]^ surface roughness,^[^ ^50^ ^]^ and even size and shape^[^ ^51^ ^]^ influence the extent of fibrosis. To mitigate these confounding effects, we tuned the modulus of PDMS control samples to be within the same order of magnitude as the PC elastomers (Figure S13, Supporting Information). Additionally, the surface topology, size, and shape of all sample discs were carefully matched (Figure S14 and S15, Supporting Information). Subcutaneous (SC) implantation studies were performed in C57BL/6 mice, with each mouse receiving implants of all four elastomer types on its back. After two weeks, the implants were retrieved for histological analysis to assess the inflammatory response in the surrounding tissue. Immunohistochemical staining was conducted for key pro‐inflammatory markers, including TNF‐α, IL‐6, CCR‐7, and IL‐17 (**Figure** **3**a,b). The results revealed that PDMS implants induced a substantial increase in inflammatory marker expression at the tissue‐implant interface compared to the mock group. In contrast, inflammation was attenuated with PURE elastomers; PURE‐C8's inflammation is slightly milder, PURE‐C16's inflammation is significantly reduced, and PURE‐C22 demonstrates the most prominent reduction.

Macrophages play a central role in coordinating the inflammatory response and are a key cell type in the conventional understanding of the foreign body response.^[^ ^52^ ^]^ However, recent studies have highlighted the crucial role of fibroblasts in this process, particularly their persistent activation into myofibroblasts, which contributes to pathological fibrosis.^[^ ^49^, ^53^ ^]^ Given this, we examined the impact of PURE elastomers on macrophage and fibroblast infiltration at the tissue‐implant interface two weeks post‐subcutaneous implantation (Figure 3c). Immunofluorescence staining for FBR‐associated cells, including myofibroblasts (α‐smooth muscle actin, α‐SMA) and macrophages (F4/80), revealed a pronounced accumulation of both cell types at the PDMS‐tissue and PURE‐C8 interfaces. In contrast, PURE‐C16 and PURE‐C22 elastomers exhibited significantly lower levels of macrophage and fibroblast recruitment, indicating reduced FBR‐related cellular overgrowth. The above results suggest that the implantation of PURE elastomers elicits a milder inflammatory response compared to PDMS, with the degree of inflammatory response being influenced by the side chain length of alkyl acrylate. The exceptionally low inflammatory response observed after the implantation of PURE‐C22 aligns well with our design strategy, where water‐driven phase reconfiguration enhances the surface prevalence of immunomodulatory THP groups, contributing to its superior immunocompatibility.

### 2.4 Assessment of Immunological and Fibrotic Responses to PURE Elastomers

**Figure 4:**
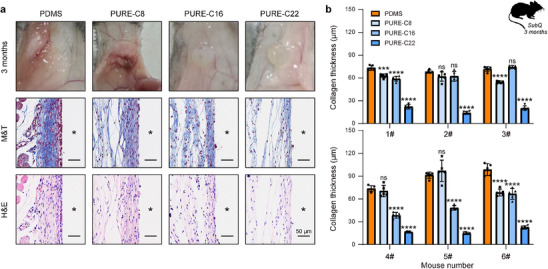
Immune responses induced by PURE elastomers after 3‐month implantation. a) Digital photographs, Masson's trichrome (M&T)‐stained, and hematoxylin and eosin (H&E)‐stained histological sections of excised tissue samples. In the histological sections, the asterisk “*” marks the original implant locations. b) Quantification of collagen capsule thickness for each mouse (*n* = 6 biologically independent replicates, mean ± s.d.). Statistical significance was determined by an unpaired, two‐tailed t‐test. ***P < 0.001, ****P < 0.0001, ns: not significant.

Next, to evaluate the degree of fibrosis, we implanted those elastomers subcutaneously in mice for three months. Representative digital photos and histological images are shown in **Figure** **4**a. Masson's trichrome staining revealed the formation of thick fibrotic capsules (68–99 µm) surrounding the PDMS implants. In contrast, implantation of PURE elastomers led to significantly reduced capsule formation, as indicated by the presence of thinner collagen layers. Among these, PURE‐C8 showed a similar fibrotic response to PDMS (54–97 µm), PURE‐C16 led to slightly thinner fibrotic capsules in some mice (38–75 µm), while PURE‐C22 showed an extremely low fibrosis response in all mice (14–23 µm) (Figure 4b). The results show that the fibrotic response caused by PURE elastomer implantation decreases with the increase of the side chain length of alkyl acrylate, which validates our previous design of the material's immunocompatibility.

### 2.5 PURE Elastomers Resist Long‐Term FBR in Mice

**Figure 5:**
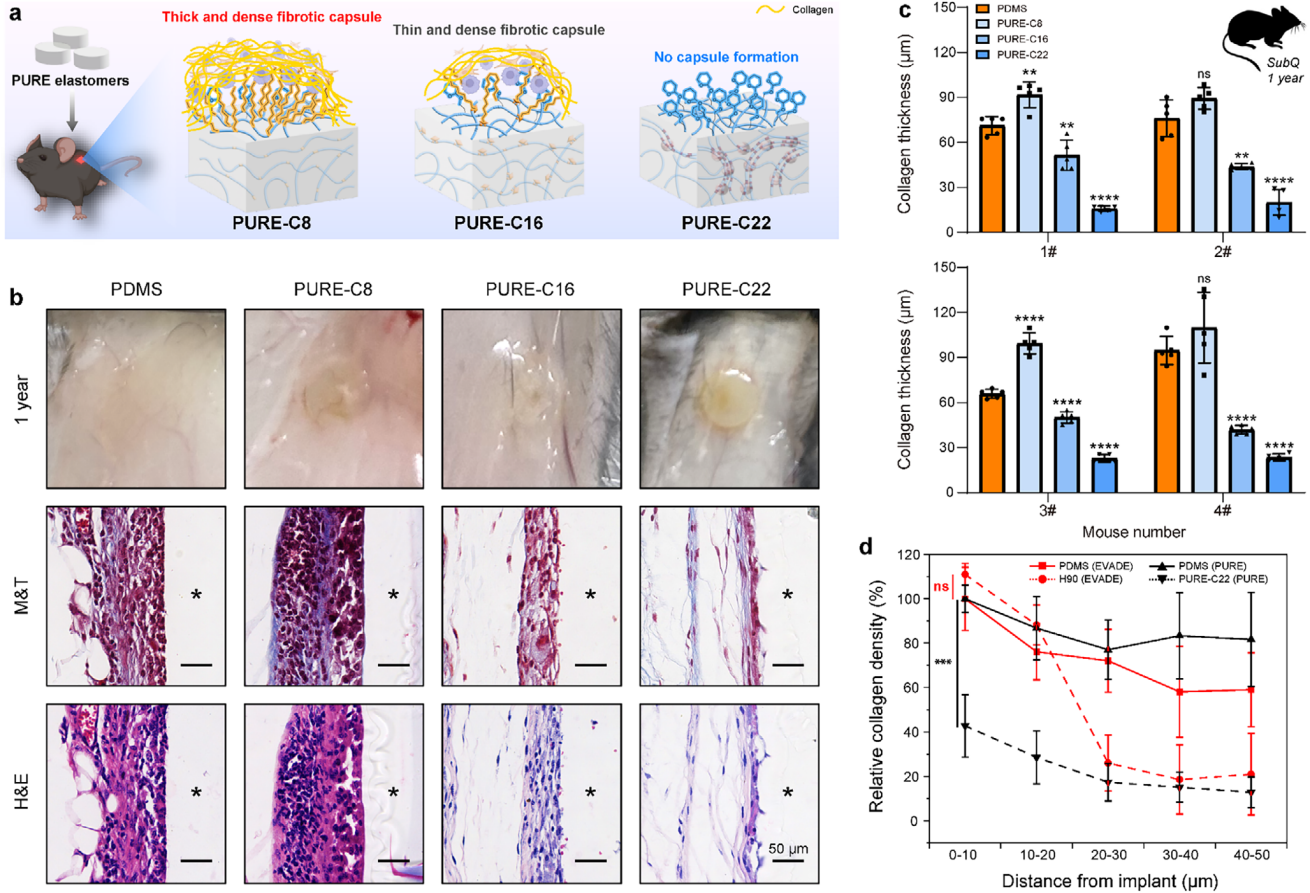
Long‐term anti‐fibrotic efficacy of PURE elastomers in mice. a) Schematic diagram of the FBR resistance by PURE elastomers. b) Digital photographs, M&T‐, and H&E‐stained histological sections of excised tissues one‐year post‐implantation in mice. In the histological sections, the asterisk “*” marks the original implant locations. c) Quantification of collagen capsule thickness for each mouse (*n* = 4 biologically independent replicates, mean ± s.d.). d) Comparison of collagen density after subcutaneous implantation of PURE and EVADE elastomers for one year (*n* = 4 mice per group). The data were normalized to the collagen density of the corresponding PDMS group. Statistical significance was determined by an unpaired, two‐tailed t‐test. **P < 0.01, ***P < 0.001, ****P < 0.0001, ns: not significant.

For implantable devices to retain their functionality in vivo, biomaterials must resist the long‐term development of fibrous capsule formation. To assess this, we conducted extended implantation tests, retrieving elastomers at six and twelve months for fibrosis analysis (**Figures** **5**a; S16, Supporting Information). Digital photos and Masson's trichrome (M&T) staining revealed that all PURE‐C22 elastomer samples maintained a distinct contour and exhibited thin, loose fibrous tissue, with no fibrous capsule observed even after one year. An apparent fibrous capsule formed around PURE‐C16, gradually becoming denser. In contrast, control PDMS elastomers and PURE‐C8 exhibited pronounced fibrosis early on, developing thick and dense collagen layers that became visually obscured at six‐ and twelve‐month post‐implantation (Figure 5b; Figure S16, Supporting Information). Analysis of capsule thickness revealed that the collagen layer surrounding PURE‐C22, PURE‐C16, and PDMS remained stable three months post‐implantation. In contrast, the collagen capsule around PURE‐C8 continued to thicken over one year (Figure 5c; Figure S16, Supporting Information). We further compared the fibrotic response of the PURE elastomer (PURE‐C22) with that of the EVADE elastomer (H90), as described in our previous work, following subcutaneous implantation in mice for one year.^[^ ^42^ ^]^ Both materials exhibited relatively thin fibrous tissue encapsulation (< 30 µm). However, collagen density analysis revealed that the PURE elastomer had a significantly lower collagen density than EVADE, with both materials normalized to the collagen density of silicone (Figure 5d). This suggests that, unlike EVADE elastomer, which forms a very thin but dense fibrotic capsule, only loose collagen fiber was generated around the PURE elastomer after one‐year implantation.

### 2.6 PURE Elastomers Resist Long‐Term FBR in NHPs

**Figure 6:**
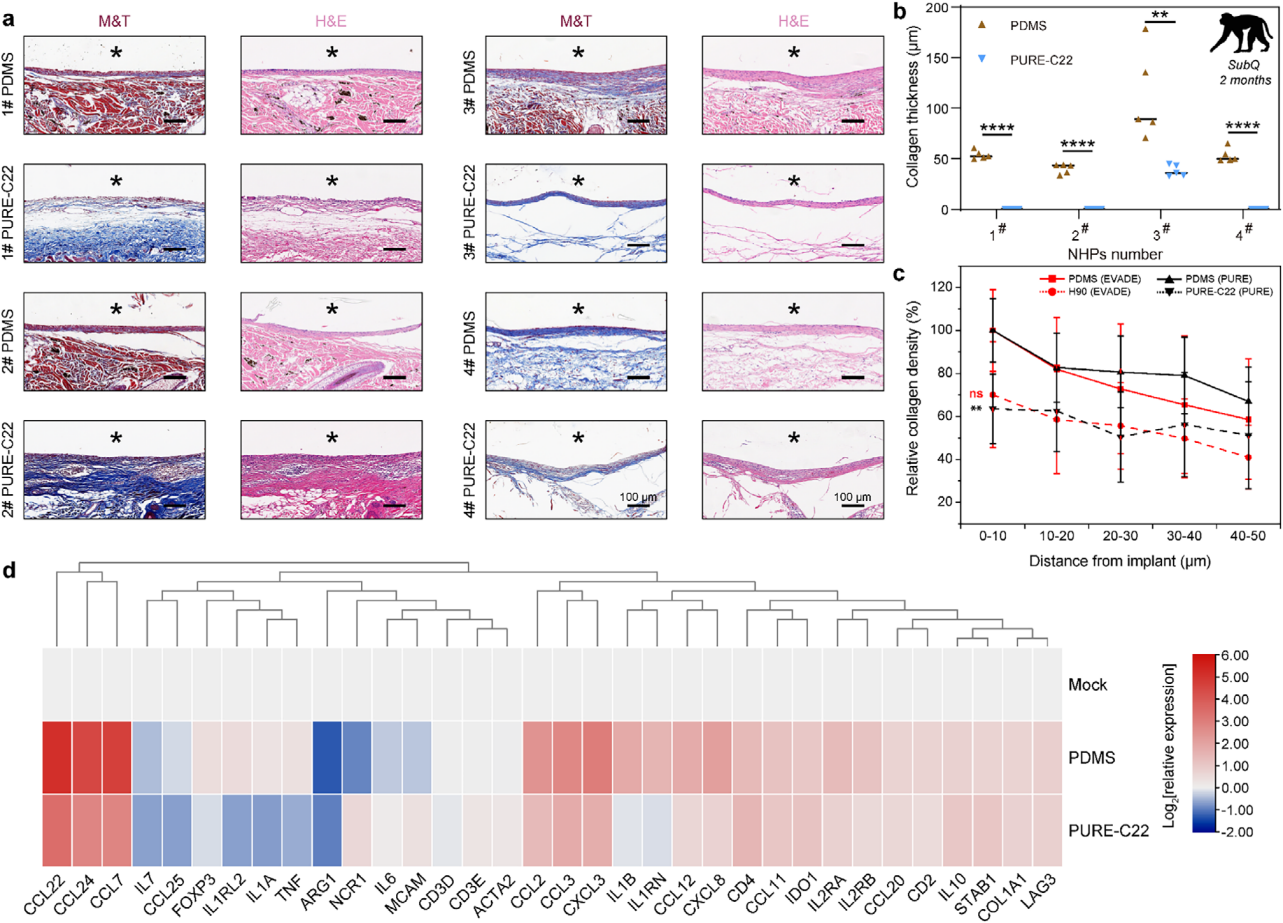
Long‐term anti‐fibrotic efficacy of PURE elastomers in NHPs. a) H&E‐ and Masson's trichrome (M&T)‐stained histological sections of excised SC tissue two months post‐implantation in NHPs. In the histological sections, the asterisk “*” marks the original implant locations. b) Quantified data on the collagen capsule thickness from M&T staining in each monkey. c) Gene expression analysis of phenotypic markers in tissues surrounding the PURE‐C22 and PDMS elastomers two months post‐implantation in NHPs. Data were normalized to the mock group and showed on a log_2_ scale (*n* = 3 monkeys per group). Statistical significance was determined by an unpaired, two‐tailed t‐test. **P < 0.01, ****P < 0.0001, ns: not significant.

To assess the translatability of our findings to a higher‐order species, we conducted subcutaneous implantation of PURE‐C22 and PDMS elastomer discs in healthy cynomolgus monkeys. Each primate received implantation of all samples in the dorsal region, and the elastomers were retrieved for examination after two months. Individual analysis of Masson sections of PDMS elastomers implanted in each monkey showed a thick fibrous response (up to 90 µm). In contrast, the PURE‐C22 material was encapsulated by thin collagen (up to 36 µm). Notably, no encapsulation was observed in three cynomolgus monkeys (**Figure** **6**a,b), suggesting that PURE elastomers can significantly reduce fibrous tissue responses in the non‐human primate (NHP) model. To further investigate the host‐mediated immune response, we performed RNA sequencing (RNA‐seq) to analyze gene expression differences between subcutaneously implanted PURE‐C22 and PDMS elastomers two months post‐implantation (Figure 6c). The results revealed significant differences in gene expression between the two groups. PURE‐C22 elastomers showed reduced expression of FBR‐associated genes, including the pro‐inflammatory cytokines *IL1A* and *IL1B*, as well as monocyte and macrophage chemoattractants C─C motif chemokine ligand 2 (*CCL2*) and C‐C motif chemokine ligand 3 (*CCL3*). T cell chemoattractants C─C motif chemokine ligand 22 (*CCL22*) and C‐C motif chemokine ligand 24 (*CCL24*) were also lower in PURE‐C22 compared to PDMS implants. In contrast, the anti‐inflammatory cytokines *IL6* and *IL10* were elevated in the PURE‐C22 group. These findings from the NHP model suggest that PURE‐C22 elastomers lead to a marked reduction in the secretion of pro‐inflammatory cytokines, provoking a host immune response that is significantly less pronounced than the response triggered by PDMS implants.

## 3 Conclusion

In summary, we present a strategy for constructing intrinsic heterogeneous surface and bulk structures within a single material, resulting in PURE elastomers that combine high elasticity with superior immunocompatibility. This is achieved through phase separation and water‐induced surface phase reconfiguration of immunoregulatory polymers. By tailoring the side chain length of the alkyl acrylates, the optimized elastomer achieves a two‐phase structure comprising alkyl chain‐rich rigid microdomains embedded in a soft immunoregulatory polymer‐rich matrix. Phase separation behavior confers exceptional bulk elasticity, characterized by high stretchability, softness, and excellent elastic recovery. More intriguingly, in a physiologically relevant moist environment, a water‐induced phase reconfiguration facilitates the migration of relatively hydrophilic, uncrosslinked immunomodulatory unit‐rich polymer chains to the elastomer surface, effectively reducing the presence of long alkyl chains associated with FBR. Consequently, the elastomer demonstrated minimal fibrotic capsule formation in rodent and non‐human primate models for up to one year and two months, respectively. This unique material design integrates high elasticity with exceptional immunocompatibility in implantable polymeric elastomers, while also providing design principles for constructing elastomers with inherent heterogeneous surface and bulk structures for other applications.

## 4 Experimental Section

### Animal Work

Mice experiments comply with the relevant regulations, and all protocols are approved by the Lab of Animal Experimental Ethical Inspection of Dr. Can Biotechnology (Zhejiang) Co., Ltd (Approval number: DRK2023150287). Wild‐type (WT) female C57BL/6 mice of 6–8 weeks in age were obtained from the animal center of Zhejiang Academy of Medical Sciences. The animals were fed a standard laboratory diet and maintained with a 12‐h light/12‐h dark cycle.

For procedures in mice, the PURE elastomers (PURE‐C8, PURE‐C16, and PURE‐C22) and PDMS sheets were cut into discs with a biophysical punch (4 mm in diameter). Elastomer samples were sterilized with 75% ethanol, washed with normal saline, and implanted subcutaneously in C57BL/6 female mice. The implantation procedure was as follows. In brief, mice were anesthetized with 3% isoflurane in oxygen, shaved, and disinfected the skin with iodine. An 8 mm longitudinal incision was made on the dorsal surface using surgical scissors to access the subcutaneous space. Then subcutaneous pockets ≈0.5 cm away from the incision were created with blunt forceps to implant the elastomer discs. After implantation, the incisions were closed using 5–0 taper‐tipped PGA absorbable sutures. Mice were monitored until recovery from anesthesia and raised for 4 h, two weeks, four weeks, three months, six months, or longer, respectively. The mice grew normally without discomfort after the implantation, and no body weight loss was observed during the entire experiment.

Analgesics were not administered during the mouse implantation surgery for the following reasons. Most commonly used analgesic drugs (e.g., Aspirin, Karlofen, and Meloxicam) operate through anti‐inflammatory mechanisms, and their use could potentially interfere with the assessment of the foreign body response (FBR) efficacy in this study. Additionally, certain non‐anti‐inflammatory analgesic drugs that could have been considered were unavailable for purchase due to local regulatory restrictions (Buprenorphine). Based on these considerations, it was decided that no analgesic drugs would be administered in this experiment.

NHP experiments comply with the relevant regulations, and all protocols are approved by the Laboratory Animal Ethics Committee of the Second Affiliated Hospital, School of Medicine, Zhejiang University (Approval number: 2023‐No.30). Cynomolgus monkeys (male; 2 to 3 years old; 2 to 3.5 kg of body weight) were obtained from Suzhou Xishan Zhongke Laboratory Animal Co. Ltd., certified by the Association for Assessment and Accreditation of Laboratory Animal Care. The animals were fed a standard laboratory diet and maintained with a 12‐h light/12‐h dark cycle.

For procedures in NHPs, the animals were anesthetized via intramuscular injection of ketamine (5 mg/kg; Gutian Pharmaceutical) and midazolam (0.2 mg kg^−1^; Ehwa Phrma) and ventilated with room air on an animal ventilator (Matrx, USA). Animals were kept on circulating warm water‐based blankets and covered during the entire procedure for body temperature maintenance. An ≈1 cm dorsal skin incision was made on the thoracic spine's left and right lateral sides. Blunt dissection was used to create a subcutaneous pocket ventrally ≈5 cm deep. PURE‐C22 and PDMS discs were placed on the left and right subcutaneous pockets. After implantation, the incisions were closed using 5–0 taper‐tipped PGA absorbable sutures. All animals received a single 50000 U kg^−1^ perioperative penicillin G benzathine/ penicillin G procaine (Combi‐Pen) injection and subcutaneous once‐daily meloxicam (0.2 mg kg^−1^ on day 1 and 0.1 mg kg^−1^ on days 2 and 3) for postsurgical pain.

### Retrieval of Tissues and Elastomers

After two weeks, one month, 3 months, six months, or longer, mice were sacrificed, and the elastomer samples and the surrounding tissue were excised and collected. The explanted samples were either fixed in 10% formaldehyde solution (for use in histology).

For NHP SubQ retrievals, animal preparation for live excision procedures was carried out at the same time as implantation (described above). Then, biopsy punches (8 mm) were used to sample the skin and SC space at two‐month retrieval time points. After retrieval, sites were closed with 5–0 taper‐tipped PGA absorbable sutures. The explanted samples were either fixed in a 10% formaldehyde solution (for use in histology) or flash‐frozen (for RNA analysis).

### Immunostaining of Tissue Sections, Microscopy, and Quantitative Image Analysis

Paraformaldehyde‐embedded tissue samples were dehydrated in graded ethanol series and embedded in paraffin. Sections of 3–5 µm in thickness were cut using a microtome, followed by deparaffinized in xylene and rehydrated in sequential baths of ethanol: distilled water. The sections were then stained with haematoxylin and eosin (cellularity) and Masson's trichrome (collagen) according to standard histological protocols. For immunohistochemistry, heat‐induced epitope retrieval was conducted using 0.1 M citrate buffer (pH 6) was performed for 15 min at 95 °C. Subsequently, the sections were further rehydrated by three washes in PBS solution containing 0.1% Tween wash buffer. Immunohistochemical staining was executed following the manufacturer's instructions (Diaminobenzidine (DAB) chromogenic reagent Kit; DAKO; catalog no. K5007). Initially, sections were blocked with peroxidase blocking solution for 10 min at room temperature. Subsequently, they underwent three washes in PBS with 0.1% Tween wash buffer. Primary antibodies directed against CCR‐7 (rabbit IgG; dilution 1:500; Abcam; catalog no. ab253187), TNF‐α (goat IgG; dilution 15 µg mL^−1^; R&D systems; catalog no. AF‐410), IL‐6 (goat IgG; dilution 15 µg mL^−1^; R&D systems; catalog no. AF‐406), and IL‐17 (rabbit IgG; dilution 1:100; Affinity; catalog no. DF6127) were used. Following this step, the sections were incubated with anti‐rabbit or anti‐goat secondary horseradish peroxidase‐conjugated antibodies goat anti‐rabbit IgG (HRP polymer; SE134; Solarbio), rabbit anti‐goat IgG (HRP polymer; GB23204; Servicebio)). For immunofluorescence staining, tissue sections were incubated in 0.1 M citrate buffer (pH 6) for 15 min at 95 °C and afterward rehydrated by three washes in TBS + 0.025% Triton X‐100 wash buffer. Sections were incubated in 10% goat serum for 60 min, followed by overnight incubation with antibodies against F4/80 (Alexa Fluor 647 polymer; rat IgG2a; dilution 5 µg mL^−1^; BioLegend; catalog no. 123 122) and α‐SMA (Cy3 polymer; mouse IgG2a; dilution 3 µg mL^−1^; Sigma; catalog no. C6198). To visualize the nuclei, 4′,6‐diamidino‐2‐phenylindole (DAPI) dihydrochloride was applied at a dilution of 1:50 (D9542; Sigma–Aldrich). Images were captured with a microscope (Nikon intensilight CHGFI) equipped with the NIS‐Elements AR software and a Virtual Slide Microscope (VS120‐S6‐W, Olympus, Japan). Regions positive (OD value) for TNF‐α, IL‐6, IL‐17, and CCR‐7 were calculated as a percentage of the positive staining in the deep or fascia‐facing capsule area, using ImageJ software. All images underwent processing with Adobe Photoshop 2023 (Adobe Systems). The contrast and brightness were enhanced consistently for all representative images used.

### RNA‐seq and Data Analysis

All appliances used were treated with diethyl pyrocarbonate (DEPC) to be RNA‐DNase free before the experiment. After implantation of elastomers, tissue samples surrounding implant materials were excised and immersed into tissue preservation solution from Baidi Biotechnology Co., Ltd. (catalog no. 3D400‐100) to preserve, using a mock group without implantation as control. RNA samples were qualified and quantified using a NanoDrop and Agilent 2100 bioanalyzer (Thermo Fisher Scientific, MA, USA). The RNA sequencing libraries were constructed and sequenced on the BGISEQ‐500 platforms. The data with a *P* value less than 0.05 with a significant difference between the PURE‐C22 and PDMS group data were counted.

### Statistics and Reproducibility

Details of the sample size and appropriate statistical test are included in the figure captions. The data are expressed as means ± s.d. The data were analyzed for statistical significance by unpaired, two‐tailed t‐test using SPSS Statistics 26.0. No adjustments were made for multiple comparisons. All in vitro and in vivo results are representative of three to six independents.

## Conflict of Interest

The authors declare no conflict of interest.

## Author Contributions

X.Z., W.C., and W.L. contributed equally to this work. P.Z. and X.Z. conceived the overall project. X.Z., W.C., W.D., and F.J. synthesized the HPEA polymers. X.Z., W.C., and Z.Z. prepared PURE elastomers. W.C. conducted mechanical tests. X.Z. conducted relevant experiments on the phase separation structure of elastomers and water‐induced phase reconfiguration. X.Z., W.C., and W.L. conducted the mouse studies. X.Z. and W.L. analyzed the histology samples. H.H. and K.Y. supervised the cynomolgus monkey study and assisted in data analysis. X.Z. and P.Z. designed the experiments, interpreted the data, and wrote the manuscript. Y.W., J.J., and P.Z. oversaw the study. All authors discussed the results and commented on the manuscript.

## Supporting information



Supporting Information

## Data Availability

The data that support the findings of this study are available from the corresponding author upon reasonable request.

## References

[1] P. Roach , D. Eglin , K. Rohde , C. C. Perry , J. Mater. Sci. Mater. Med. 2007, 18, 1263. 17443395 10.1007/s10856-006-0064-3

[2] J. Wu , J. Deng , G. Theocharidis , T. L. Sarrafian , L. G. Griffiths , R. T. Bronson , A. Veves , J. Chen , H. Yuk , X. Zhao , Nature 2024, 630, 360. 38778109 10.1038/s41586-024-07426-9 PMC11168934

[3] C. C. Schreib , M. I. Jarvis , T. Terlier , J. Goell , S. Mukherjee , M. D. Doerfert , T. A. Wilson , M. Beauregard , K. N. Martins , J. Lee , L. Sanchez Solis , E. Vazquez , M. A. Oberli , B. W. Hanak , M. Diehl , I. Hilton , O. Veiseh , Adv. Mater. 2023, 35, 2205709. 10.1002/adma.202205709 PMC10309593 36871193

[4] J. M. Anderson , A. Rodriguez , D. T. Chang , Semin. Immunol. 2008, 20, 86. 18162407 10.1016/j.smim.2007.11.004 PMC2327202

[5] Y. K. Kim , E. Y. Chen , W. F. Liu , J. Mater. Chem. B 2016, 4, 1600. 32263014 10.1039/c5tb01605c

[6] O. Veiseh , A. J. Vegas , Adv. Drug Delivery Rev. 2019, 144, 148. 10.1016/j.addr.2019.08.010 PMC6774350 31491445

[7] H. Hao , Y. Xue , Y. Wu , C. Wang , Y. Chen , X. Wang , P. Zhang , J. Ji , Bioact. Mater. 2023, 28, 1. 37214260 10.1016/j.bioactmat.2023.04.022 PMC10192934

[8] Y. Chen , Y. Chen , W. Cao , J. Wang , P. Zhang , J. Ji , Langmuir 2025, 41, 2591. 39848705 10.1021/acs.langmuir.4c04364

[9] H. Ji , K. Yu , S. Abbina , L. Xu , T. Xu , S. Cheng , S. Vappala , S. A. Arefi , M. M. Rana , I. Chafeeva , M. Drayton , K. Gonzalez , Y. Liu , D. Grecov , E. M. Conway , W. Zhao , C. Zhao , J. N. Kizhakkedathu , Nat. Mater. 2024, 24, 626. 39533064 10.1038/s41563-024-02046-0 PMC11961369

[10] X. Wang , Y. Yin , J. Wang , H. Yu , Q. Tang , Z. Chen , G. Fu , K. Ren , J. Ji , L. Yu , Adv. Sci. 2024, 11, 2401301. 10.1002/advs.202401301 PMC11187865 38544484

[11] Q. Gao , X. Li , W. Yu , F. Jia , T. Yao , Q. Jin , J. Ji , ACS Appl. Mater. Interfaces 2019, 12, 2999. 31845798 10.1021/acsami.9b19335

[12] J. Wang , Y. Xue , J. Liu , M. Hu , H. Zhang , K. Ren , Y. Wang , J. Ji , Research 2020, 2020, 1458090. 32885169 10.34133/2020/1458090 PMC7455884

[13] J. Wang , X. Y. Li , H. L. Qian , X. W. Wang , Y. X. Wang , K. F. Ren , J. Ji , Adv. Mater. 2024, 36, 2310216. 10.1002/adma.202310216 38237136

[14] X. Zhou , H. Hao , Y. Chen , W. Cao , Z. Zhu , Y. Ni , Z. Liu , F. Jia , Y. Wang , J. Ji , Z. Peng , Bioact. Mater. 2024, 34, 482. 38292409 10.1016/j.bioactmat.2024.01.006 PMC10827492

[15] D. Dong , C. Tsao , H.‐C. Hung , F. Yao , C. Tang , L. Niu , J. Ma , J. MacArthur , A. Sinclair , K. Wu , P. Jain , M. R. Hansen , D. Ly , S. G. Tang , T. M. Luu , P. Jain , S. Jiang , Sci. Adv. 2021, 7, abc5442. 10.1126/sciadv.abc5442 PMC7775767 33523839

[16] D. Zhang , Q. Chen , C. Shi , M. Chen , K. Ma , J. Wan , R. Liu , Adv. Funct. Mater. 2020, 31, 2007226.

[17] X. Zhou , Y. Wang , J. Ji , P. Zhang , Adv. Healthcare Mater. 2024, 13, 2304478. 10.1002/adhm.202304478 38666550

[18] Z. Liu , X. Zhou , Y. Chen , Y. Ni , Z. Zhu , W. Cao , K. Chen , Y. Yan , J. Ji , P. Zhang , Biomater. Sci. 2024, 12, 468. 38086632 10.1039/d3bm01783d

[19] X. Zhou , W. Cao , Y. Chen , Z. Zhu , Y. Chen , Y. Ni , Z. Liu , F. Jia , Z. Lu , Y. Ye , H. Han , K. Yao , W. Liu , X. Wei , S. Chen , Y. Wang , J. Ji , P. Zhang , Adv. Sci. 2024, 11, 2308077. 10.1002/advs.202308077 PMC11040334 38403462

[20] E. Zhang , Y. Shi , X. Han , H. Zhu , B. Song , C. Yang , Z. Cao , Nat. Biomed. Eng. 2023, 8, 1197. 37884794 10.1038/s41551-023-01108-z

[21] Q. Liu , X. Wang , A. Chiu , W. Liu , S. Fuchs , B. Wang , L. H. Wang , J. Flanders , Y. Zhang , K. Wang , J. M. Melero‐Martin , M. Ma , Adv. Mater. 2021, 33, 2102852. 10.1002/adma.202102852 PMC8487957 34363254

[22] L. Chung , D. R. Maestas , A. Lebid , A. Mageau , G. D. Rosson , X. Wu , M. T. Wolf , A. J. Tam , I. Vanderzee , X. Wang , J. I. Andorko , H. Zhang , R. Narain , K. Sadtler , H. Fan , D. Čiháková , C. J. L. Saux , F. Housseau , D. M. Pardoll , J. H. Elisseeff , Sci. Transl. Med. 2020, 12, aax3799. 10.1126/scitranslmed.aax3799 PMC7219543 32295900

[23] T. R. Kyriakides , H. J. Kim , C. Zheng , L. Harkins , W. Tao , E. Deschenes , Biomed. Mater. 2022, 17, 022007. 10.1088/1748-605X/ac5574 PMC9159526 35168213

[24] M. Asadikorayem , P. Weber , F. Surman , A. Puiggalí‐Jou , M. Zenobi‐Wong , Adv. Healthcare Mater. 2025, 14, 2402890. 10.1002/adhm.202402890 PMC11730820 39498680

[25] H. Yan , C. Seignez , M. Hjorth , B. Winkeljann , M. Blakeley , O. Lieleg , M. Phillipson , T. Crouzier , Adv. Funct. Mater. 2019, 29, 1902581.

[26] L. Zhang , Z. Cao , T. Bai , L. Carr , J. R. Ella‐Menye , C. Irvin , B. D. Ratner , S. Jiang , Nat. Biotechnol. 2013, 31, 553. 23666011 10.1038/nbt.2580

[27] H. M. Rostam , L. E. Fisher , A. L. Hook , L. Burroughs , J. C. Luckett , G. P. Figueredo , C. Mbadugha , A. C. K. Teo , A. Latif , L. Kämmerling , M. Day , K. Lawler , D. Barrett , S. Elsheikh , M. Ilyas , D. A. Winkler , M. R. Alexander , A. M. Ghaemmaghami , Matter 2020, 2, 1564.

[28] W. J. Jeang , B. M. Wong , Y. Zhao , R. S. Manan , A. L. Jiang , S. Bose , E. Collins , P. McMullen , J. G. Rosenboom , S. Lathwal , R. Langer , D. G. Anderson , Adv. Mater. 2024, 37, 2414743. 10.1002/adma.202414743 PMC12911169 39722171

[29] H. He , X. Zhou , Y. Lai , R. Wang , H. Hao , X. Shen , P. Zhang , J. Ji , Nat. Commun. 2025, 16, 3004. 40148278 10.1038/s41467-025-58171-0 PMC11950410

[30] Y. Yan , X. Zhou , Z. Liu , Z. Zhu , W. Cao , K. Chen , J. Ji , P. Zhang , Langmuir 2025, 41, 9131. 40145243 10.1021/acs.langmuir.5c00868

[31] M. Zhou , Z. Wang , M. Li , Q. Chen , S. Zhang , J. Wang , Biomaterials 2025, 317, 123010. 39724767 10.1016/j.biomaterials.2024.123010

[32] X. Xie , J. C. Doloff , V. Yesilyurt , A. Sadraei , J. J. McGarrigle , M. Omami , O. Veiseh , S. Farah , D. Isa , S. Ghani , I. Joshi , A. Vegas , J. Li , W. Wang , A. Bader , H. H. Tam , J. Tao , H. J. Chen , B. Yang , K. A. Williamson , J. Oberholzer , R. Langer , D. G. Anderson , Nat. Biomed. Eng. 2018, 2, 894. 30931173 10.1038/s41551-018-0273-3 PMC6436621

[33] S. Bose , L. R. Volpatti , D. Thiono , V. Yesilyurt , C. McGladrigan , Y. Tang , A. Facklam , A. Wang , S. Jhunjhunwala , O. Veiseh , J. Hollister‐Lock , C. Bhattacharya , G. C. Weir , D. L. Greiner , R. Langer , D. G. Anderson , Nat. Biomed. Eng. 2020, 4, 814. 32231313 10.1038/s41551-020-0538-5 PMC8051527

[34] D. Chan , C. L. Maikawa , A. I. d'Aquino , S. S. Raghavan , M. L. Troxell , E. A. Appel , J. Biomed. Mater. Res., Part A 2023, 111, 910. 10.1002/jbm.a.37521 PMC10161736 36861657

[35] S. Mukherjee , B. Kim , L. Y. Cheng , M. D. Doerfert , J. Li , A. Hernandez , L. Liang , M. I. Jarvis , P. D. Rios , S. Ghani , I. Joshi , D. Isa , T. Ray , T. Terlier , C. Fell , P. Song , R. N. Miranda , J. Oberholzer , D. Y. Zhang , O. Veiseh , Nat. Biomed. Eng. 2023, 7, 867. 37106151 10.1038/s41551-023-01016-2 PMC10593184

[36] Q. Liu , A. Chiu , L. H. Wang , D. An , M. Zhong , A. M. Smink , B. J. de Haan , P. de Vos , K. Keane , A. Vegge , E. Y. Chen , W. Song , W. F. Liu , J. Flanders , C. Rescan , L. G. Grunnet , X. Wang , M. Ma , Nat. Commun. 2019, 10, 5262. 31748525 10.1038/s41467-019-13238-7 PMC6868136

[37] Z. Wang , M. Zhou , M. Li , J. Li , S. Zhang , J. Wang , Bioact. Mater. 2024, 41, 127. 39131628 10.1016/j.bioactmat.2024.07.006 PMC11314893

[38] H. Wang , H. Li , Y. Wu , J. Yang , W. Liu , Sci. China Technol. Sci. 2019, 62, 569.

[39] Q. Liu , A. Chiu , L. Wang , D. An , W. Li , E. Y. Chen , Y. Zhang , Y. Pardo , S. P. McDonough , L. Liu , W. F. Liu , J. Chen , M. Ma , Biomaterials 2020, 230, 119640. 31791840 10.1016/j.biomaterials.2019.119640

[40] H. Wang , Y. Hu , D. Lynch , M. Young , S. Li , H. Cong , F. J. Xu , G. Cheng , ACS Appl. Mater. Interfaces 2018, 10, 37609. 30335927 10.1021/acsami.8b10450

[41] X. Zhou , W. Cao , Y. Chen , Z. Zhu , Y. Lai , Z. Liu , F. Jia , Z. Lu , H. Han , K. Yao , Y. Wang , J. Ji , P. Zhang , Acta Biomater. 2024, 185, 226. 38972625 10.1016/j.actbio.2024.06.047

[42] X. Zhou , Z. Lu , W. Cao , Z. Zhu , Y. Chen , Y. Ni , Z. Liu , F. Jia , Y. Ye , H. Han , K. Yao , W. Liu , Y. Wang , J. Ji , P. Zhang , Nat. Commun. 2024, 15, 7526. 39214984 10.1038/s41467-024-52023-z PMC11364871

[43] S. Riemer , S. Prévost , M. Dzionara , M.‐S. Appavou , R. Schweins , M. Gradzielski , Polymer 2015, 70, 194.

[44] X. Wang , S. Zhan , Z. Lu , J. Li , X. Yang , Y. Qiao , Y. Men , J. Sun , Adv. Mater. 2020, 32, 2005759. 10.1002/adma.202005759 33175420

[45] H. C. Hung , P. Jain , P. Zhang , F. Sun , A. Sinclair , T. Bai , B. Li , K. Wu , C. Tsao , E. J. Liu , H. S. Sundaram , X. Lin , P. Farahani , T. Fujihara , S. Jiang , Adv. Mater. 2017, 29, 1700617. 10.1002/adma.201700617 28620970

[46] D. Zhang , Q. Chen , Y. Bi , H. Zhang , M. Chen , J. Wan , C. Shi , W. Zhang , J. Zhang , Z. Qiao , J. Li , S. Chen , R. Liu , Nat. Commun. 2021, 12, 5327. 34493717 10.1038/s41467-021-25581-9 PMC8423817

[47] K. T. Huang , P. S. Hsieh , L. G. Dai , C. J. Huang , J. Mater. Chem. B 2020, 8, 7390. 32657299 10.1039/d0tb01163k

[48] H. Xiang , X. Li , B. Wu , S. Sun , P. Wu , Adv. Mater. 2023, 35, 2209581. 10.1002/adma.202209581 36670074

[49] N. Noskovicova , R. Schuster , S. van Putten , M. Ezzo , A. Koehler , S. Boo , N. M. Coelho , D. Griggs , P. Ruminski , C. A. McCulloch , B. Hinz , Nat. Biomed. Eng. 2021, 5, 1437. 34031559 10.1038/s41551-021-00722-z

[50] J. C. Doloff , O. Veiseh , R. de Mezerville , M. Sforza , T. A. Perry , J. Haupt , M. Jamiel , C. Chambers , A. Nash , S. Aghlara‐Fotovat , J. L. Stelzel , S. J. Bauer , S. Y. Neshat , J. Hancock , N. A. Romero , Y. E. Hidalgo , I. M. Leiva , A. M. Munhoz , A. Bayat , B. M. Kinney , H. C. Hodges , R. N. Miranda , M. W. Clemens , R. Langer , Nat. Biomed. Eng. 2021, 5, 1115. 34155355 10.1038/s41551-021-00739-4

[51] O. Veiseh , J. C. Doloff , M. Ma , A. J. Vegas , H. H. Tam , A. R. Bader , J. Li , E. Langan , J. Wyckoff , W. S. Loo , S. Jhunjhunwala , A. Chiu , S. Siebert , K. Tang , J. Hollister‐Lock , S. Aresta‐Dasilva , M. Bochenek , J. Mendoza‐Elias , Y. Wang , M. Qi , D. M. Lavin , M. Chen , N. Dholakia , R. Thakrar , I. Lacik , G. C. Weir , J. Oberholzer , D. L. Greiner , R. Langer , D. G. Anderson , Nat. Mater. 2015, 14, 643. 25985456 10.1038/nmat4290 PMC4477281

[52] J. C. Doloff , O. Veiseh , A. J. Vegas , H. H. Tam , S. Farah , M. Ma , J. Li , A. Bader , A. Chiu , A. Sadraei , S. Aresta‐Dasilva , M. Griffin , S. Jhunjhunwala , M. Webber , S. Siebert , K. Tang , M. Chen , E. Langan , N. Dholokia , R. Thakrar , M. Qi , J. Oberholzer , D. L. Greiner , R. Langer , D. G. Anderson , Nat. Mater. 2017, 16, 671. 28319612 10.1038/nmat4866 PMC5445003

[53] F. S. Younesi , A. E. Miller , T. H. Barker , F. M. V. Rossi , B. Hinz , Nat. Rev. Mol. Cell Biol. 2024, 25, 617. 38589640 10.1038/s41580-024-00716-0

