# Supplementary material for: Immunocompatible Elastomer With Enhanced Fibrous Capsule‐Resistance and Elasticity by Water‐Induced Surface Phase Reconfiguration
Source: Adv Sci (Weinh). 2025 Jun 25;12(35):e04801. doi: 10.1002/advs.202504801 (PMC12463074; doi:10.1002/advs.202504801)
Supplement: Supplementary file 1 — Supporting Information [file ADVS-12-e04801-s001.docx]

Supporting Information

**Immunocompatible Elastomer with Enhanced Fibrous Capsule-resistance and Elasticity by Water-induced Surface Phase Reconfiguration**

*Xianchi Zhou, Wenzhong Cao, Weifeng Liu, Wenbin Dai, Zihao Zhu, Fan Jia, Haijie Han, Ke Yao, Youxiang Wang, Jian Ji and Peng Zhang**

**Experimental Procedures**

**Materials and reagents.** 2-Hydroxyethyl acrylate (HEA), 2,3-Dihydropyran, Amberlyst H15, 1,4-Butanediol diacrylate, *n*-Octyl acrylate (C8), Hexadecyl acrylate (C16), Docosyl acrylate (C22), Azobisisobutyronitrile (AIBN), and Irgacure 819 were supplied by Aladdin Reagent Co., Ltd. Phosphate buffered saline (PBS, 10 mM phosphate, 138 mM sodium chloride, 2.7 mM potassium chloride, pH 7.4) was obtained from Sangon Biotech Co., Ltd. The silicone elastomer Sylgard 184 was obtained from Dow Corning (catalog no. 3097358-1004).

**Synthesis of HPEA Monomers.** 2-((Tetrahydro-2H-pyran-2-yl)oxy)ethyl acrylate (HPEA) was synthesized as follows. To a stirred solution of HEA (4.64 g, 1.0 eq.) in 50 mL of hexane was added Amberlyst H15 (1 g) and 2,3-dihydropyran (3.70 g, 1.1 eq.). The reaction contents were stirred in the dark for 2 h, filtered, and concentrated. Then washed the organic phase with deionized water, dried the organic phase with anhydrous MgSO_4_, and concentrated to give desired monomer as yellow liquid in 82% yield (6.56 g). ^1^H NMR spectra were collected on a Bruker AVANCE III 400 spectrometer at 400 MHz, using CDCl_3_ as the solvent. ^1^H NMR chemical shifts were referenced to the resonance for residual protonated solvent (*δ* 0.00 for TMS in CDCl_3_). ^1^H NMR (400 MHz, CDCl_3_) *δ* 6.50 – 6.39 (m, 1H), 6.23 – 6.11 (m, 1H), 5.91 – 5.80 (m, 1H), 4.66 (t, *J* = 3.6 Hz, 1H), 4.35 (qdd, *J* = 11.8, 5.9, 3.5 Hz, 2H), 3.96 (ddd, *J* = 11.5, 6.1, 3.5 Hz, 1H), 3.91 – 3.82 (m, 1H), 3.80 – 3.63 (m, 1H), 3.57 – 3.47 (m, 1H), 1.92 – 1.67 (m, 2H), 1.67 – 1.54 (m, 2H), 1.53 (tdd, *J* = 8.3, 4.2, 2.2 Hz, 2H).

**Fabrication and characterization of PURE elastomers**. The PURE elastomers were fabricated by radical bulk copolymerization in glass molds with the desired shape. For chemically crosslinked elastomers, a reaction mixture containing HPEA monomer, 1,4-butanediol diacrylate crosslinker, and 0.5 wt% (relative to the total weight of monomers) of photoinitiator Irgacure 819 was photo-polymerized using glass slides separated by a 1 mm thick polytetrafluoroethylene (PTFE) spacer. The amount of crosslinker added is 0.5 mol% (PURE-0.5%), 1.0 mol% (PURE-1.0%), and 1.5 mol% (PURE-1.5%). Physically crosslinked elastomers were synthesized using a similar procedure, where 1,4-butanediol diacrylate is replaced with 10 mol% (relative to the total mole of monomers) of *n*-octyl acrylate (PURE-C8), hexadecyl acrylate (PURE-C16), and docosahexadecyl acrylate (PURE-C22). The reaction proceeded under UV irradiation (365 nm) at about 180 mW/cm^2^ for 10 min. After polymerization, the elastomer samples were soaked in sterile PBS buffer (pH 7.4) for 2 days, followed by dried under vacuum at room temperature for further tests. PDMS elastomers were prepared and used as controls, with the ratio of prepolymer and curing agent adjusted to ensure a similar tensile modulus to PURE elastomers. The mixture was degassed and poured into plastic dishes at a fixed volume, resulting in a PDMS layer with a thickness of ~1 mm. The PDMS layer was cured at 80°C for 2 h, peeled, and subsequently cleaned with ethanol and dried.

*DSC.* The T_g_s of PURE elastomers were measured by DSC (Q20, TA instruments, New Castle, DE, USA) at a scanning range of −70 to 150°C with a heating/cooling rate of 10°C min^−1^ and a nitrogen flow rate of 50 mL min^−1^. During the test, each sample was heated to 150°C, kept at 150°C for 3 min to eliminate thermal history, cooled to −70°C, and then heated to 150°C again. T_g_ was taken as the inflection of the DSC curve.

*XRD.* The crystallization behavior of PURE elastomers was measured by Powder X-ray Diffractometer (Rigaku Ultima IV) with Cu *Kα* radiation in the 2θ range 3−60° with a step size of 0.02° (40 mA, 40 kV).

*Mechanical Tests.* Tensile tests were measured on a tensile-compressive mechanical tester (Zwick/Roell Z020) with a 500 N load cell at room temperature, respectively. For tensile tests, PURE elastomers were cut from the elastomer sheet into a dumbbell shape with an initial gauge length of 12 mm and a width of 2 mm. The stretch rate was 10 mm min^-1^, and the nominal stress and strain were recorded. Young’s modulus of the elastomer was calculated from the initial slope of the tensile stress-strain curve with a strain below 10%. Average data were acquired by testing three specimens for each sample. The stress-strain curve of the PURE elastomers in cyclic stress-strain tests (100% strain) in successive stretching was conducted using the same size samples as mentioned above.

*Rheological experiments*. The rheological behavior of elastomers was carried out using a TA ARES-G2 rheometer with an 8 mm parallel plate geometry.

*Non-contact profilometry.* Samples with a diameter of 4 mm were first cleaned with 99% isopropyl alcohol. They were then positioned on an anti-vibration table beneath the ×20 lens of a non-contact profilometer (KEYENCE VR-5200). The samples were carefully adjusted to ensure a 90° angle between the surface of interest and the lens, followed by focusing. Smooth surfaces were captured in automatic mode; for unknown topographies, the upper and lower z-axis limits were manually adjusted. In the confocal program’s visualization mode, the z-limits were set such that both focusing extremes appeared completely black in the observed image. The visualization mode was then switched to microscope mode, with automatic brightness adjustment to prevent overexposure, ensuring no areas appeared red. Images and measurements were subsequently recorded for each sample, and the data were analyzed.

**Analysis of compositional drift by varying conversion**. A set of 9 individual batches of HPEA-alkyl acrylate polymers were made and brought to various conversions to examine the possibility of compositional drift. AIBN was recrystallized in ethanol prior to use. HPEA monomer is newly synthesized for immediate use without the addition of polymerization inhibitors. Alkyl acrylates were used as received. Reaction times were varied in order to target approximate conversions. The specific reactants and their feeding ratios are listed in Supplementary Table 4.

Flat-bottomed 20 mL glass bottles were each charged with reactants and dissolved with 10 mL *N*,*N*-dimethylformamide (DMF). Each reaction mixture was degassed by bubbling argon gas for 20 min and then use an argon gas balloon to maintain the reaction system in an argon atmosphere. The bottles were held in a 65^o^C oven for the corresponding time. An aliquot of 50 μL of the sample is mixed with 550 μL of deuterated reagent, and the mixture is subsequently analyzed using NMR spectroscopy.

Individual monomer conversion was calculated from ^1^H NMR using the polymerization solvent (DMF at 7.88 ppm) as the internal standard. Monomers C8 (8.12 ppm), C16 (8.13 ppm), C22 (8.14 ppm), and HPEA (6.21-6.35 ppm) could be resolved individually. Global monomer conversion was calculated from the individual monomer conversions together with the total monomer composition. The individual conversions were plotted against the total conversions for all samples.

**Polarized optical microscopic (POM)**. Images of elastomer samples under dry conditions were taken on a polarizing microscope (CX40P, SOPTOP) in the presence of a 530 nm tint plate. For wet samples, elastomers were soaked in PBS for 5 min and then the surface of the sample was dried with a lint-free paper and observed using the polarizing microscope.

**Small-angle X-ray scattering (SAXS)**. SAXS curves and 2D-SAXS patterns of elastomers were performed on a Xeuss 2.0 system (Xenocs SA, France) equipped with a semiconductor detector (Pilatus 100 K, DECTRIS, Switzerland) attached to a multilayer-focused Cu Kα X-ray source (GeniX3D Cu ULD, Xenocs SA, France). The

wavelength of the X-ray radiation was 1.5418 Å. The sample-to-detector distance was 360 mm. The exposure time of the elastomers to the X-ray source was 5 min. The authors would like to thank Meifang Wang from Shiyanjia Lab (www.shiyanjia.com) for providing assistance with the SAXS tests.

**Fourier Transform Infrared Spectroscopy (FTIR)**. The phase reconfiguration behaviour of the PURE elastomers was analyzed by FTIR under dry conditions using a Thermo Nicolet 6700 FT-IR Spectrometer with a Smart iTR Attenuated Total Reflectance (ATR). The wavenumber ranged from 500 to 4,000 cm^−1^ with 32 scans. For wet samples, elastomers were soaked in PBS for 5 min and then the surface of the sample was dried with a lint-free paper and tested.

**Supplementary Figures**

**Supplementary Fig. 1 | Schematic diagram of the chemical synthesis of HPEA monomer.**


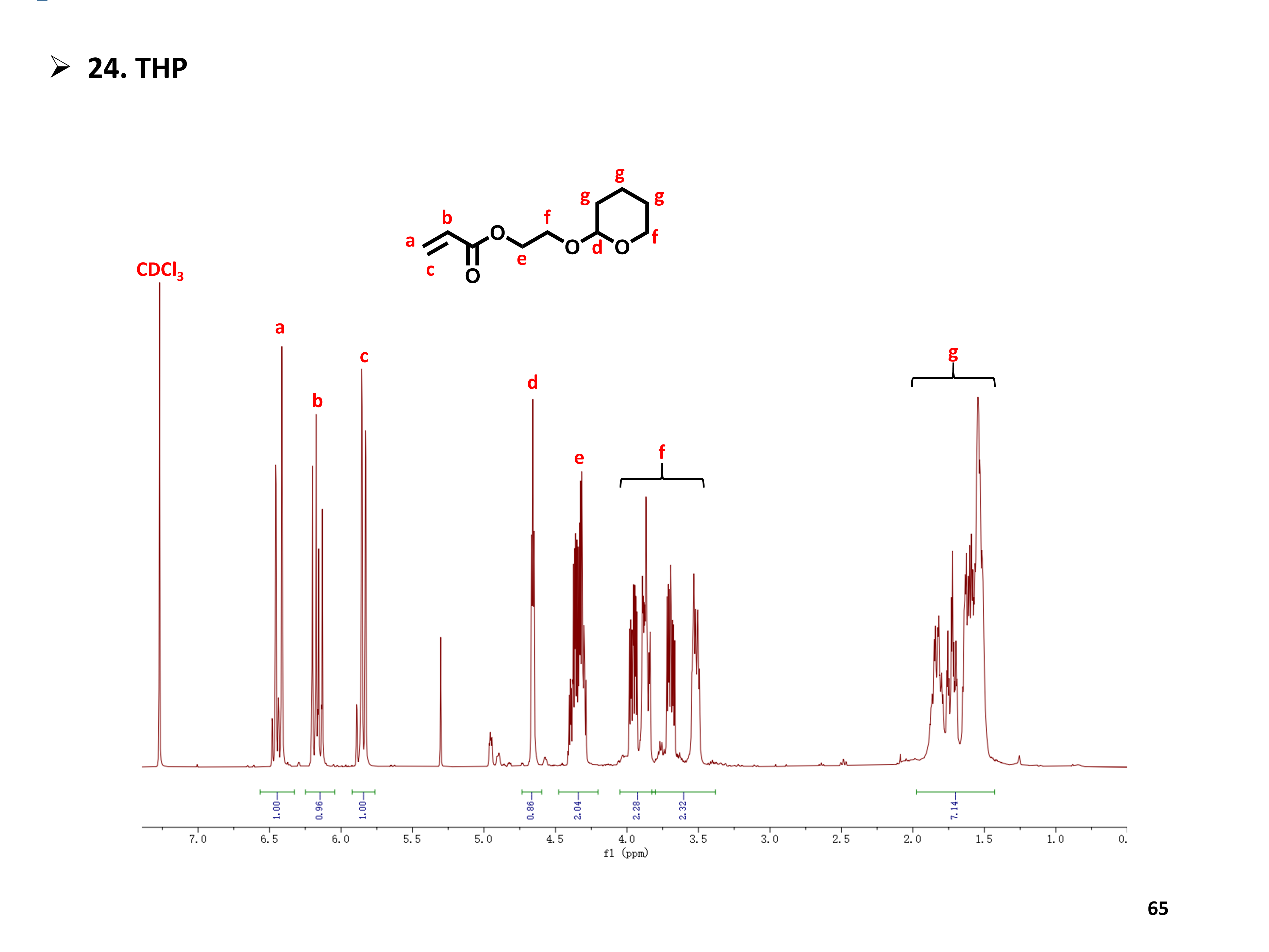


**Supplementary Fig. 2 | NMR spectrum of HPEA monomer.** ^1^H NMR (400 MHz, CDCl_3_) spectrum of HPEA monomer.


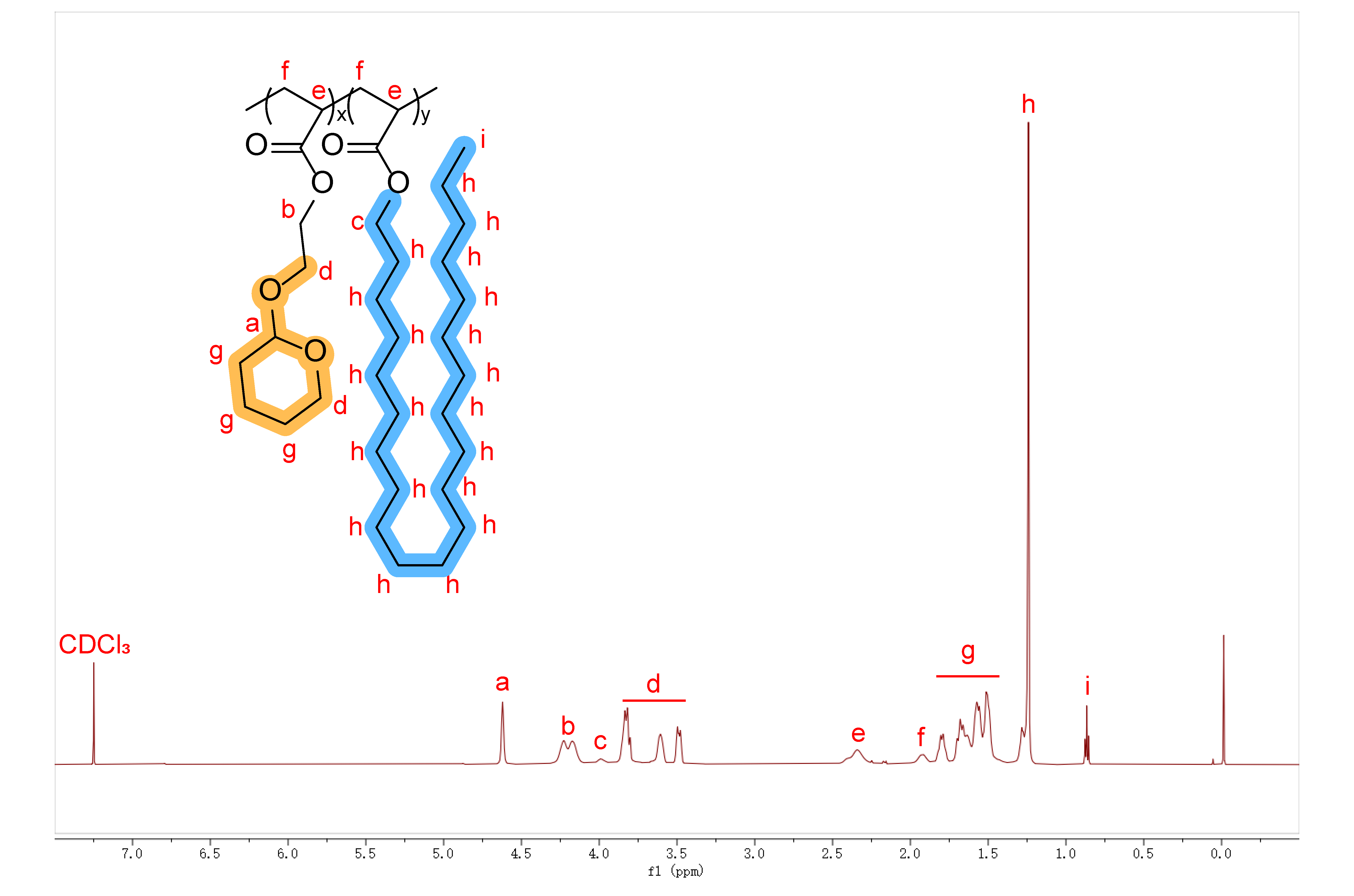


**Supplementary Fig. 3 | ^1^H NMR (400 MHz, CDCl_3_) spectrum of PURE-C22 elastomer.**


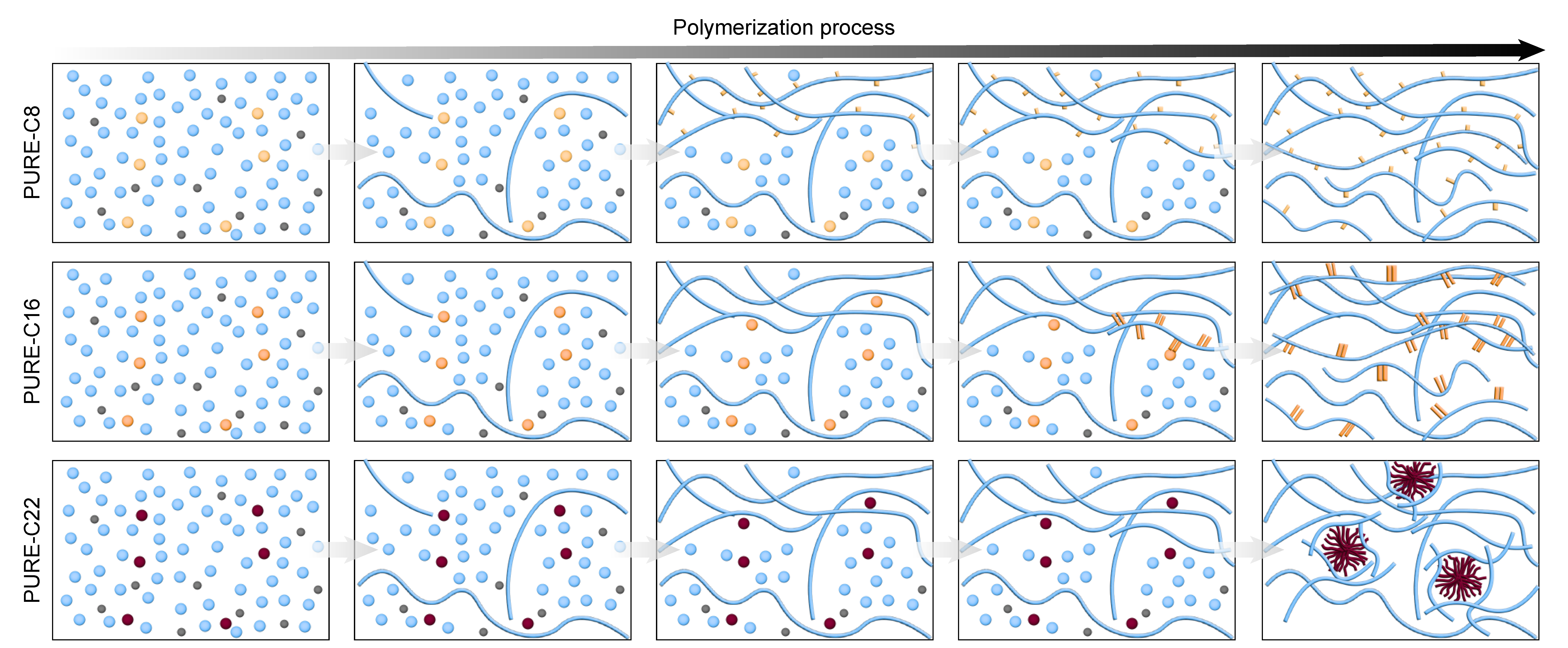


**Supplementary Fig. 4 | Proposed mechanisms of elastomer polymerization kinetics.**


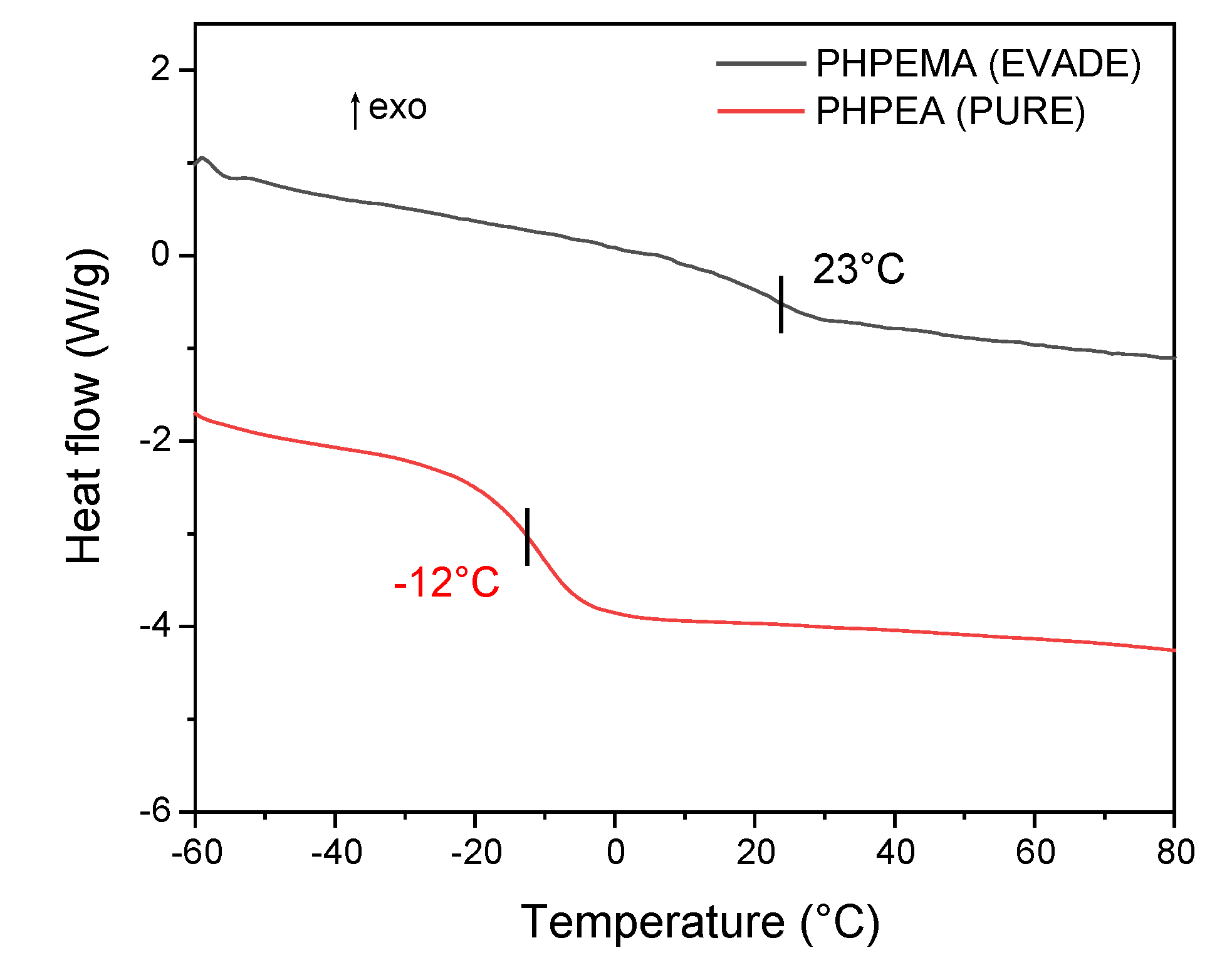


**Supplementary Fig. 5 | DSC curves of HPEMA (EVADE) and HPEA (PURE) homopolymer.** During the test, each sample was heated to 150°C, kept at 150°C for 3 min to eliminate thermal history, cooled to −70°C then heated to 150°C again.


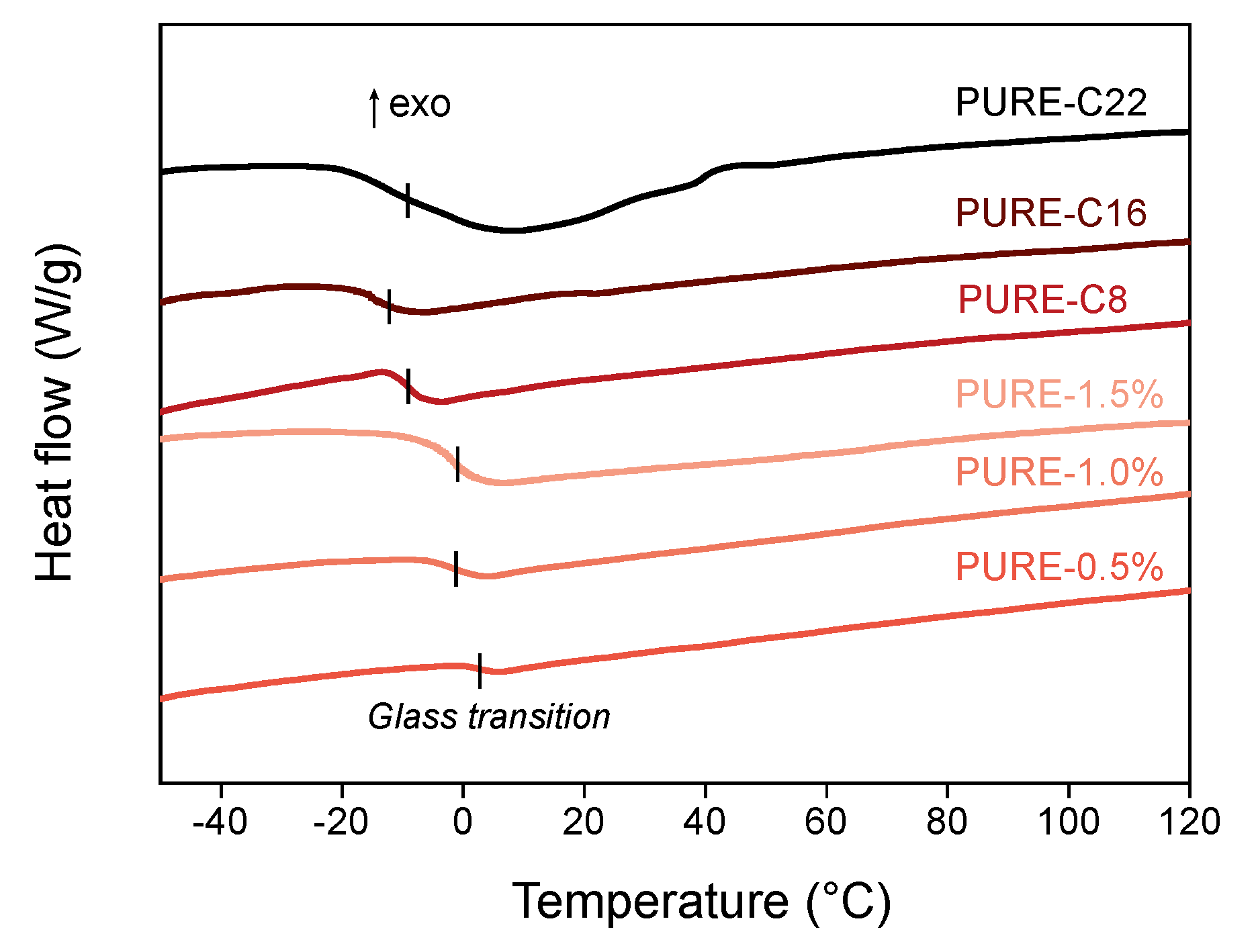


**Supplementary Fig. 6 | DSC curves of PURE elastomers.** During the test, each sample was heated to 150°C, kept at 150°C for 3 min to eliminate thermal history, cooled to −70°C then heated to 150°C again.


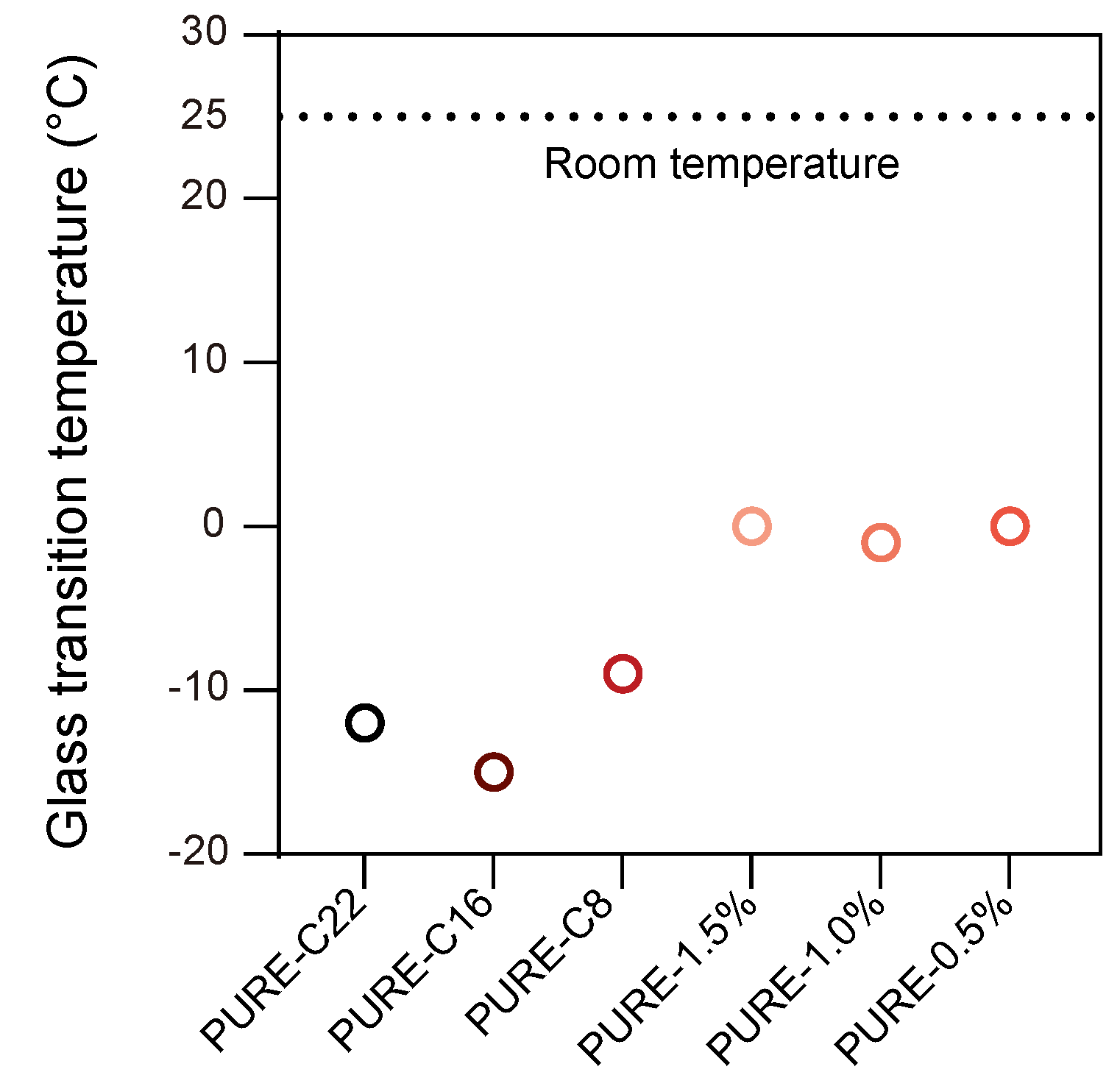


**Supplementary Fig. 7 | The glass transition temperature of PURE elastomers.**


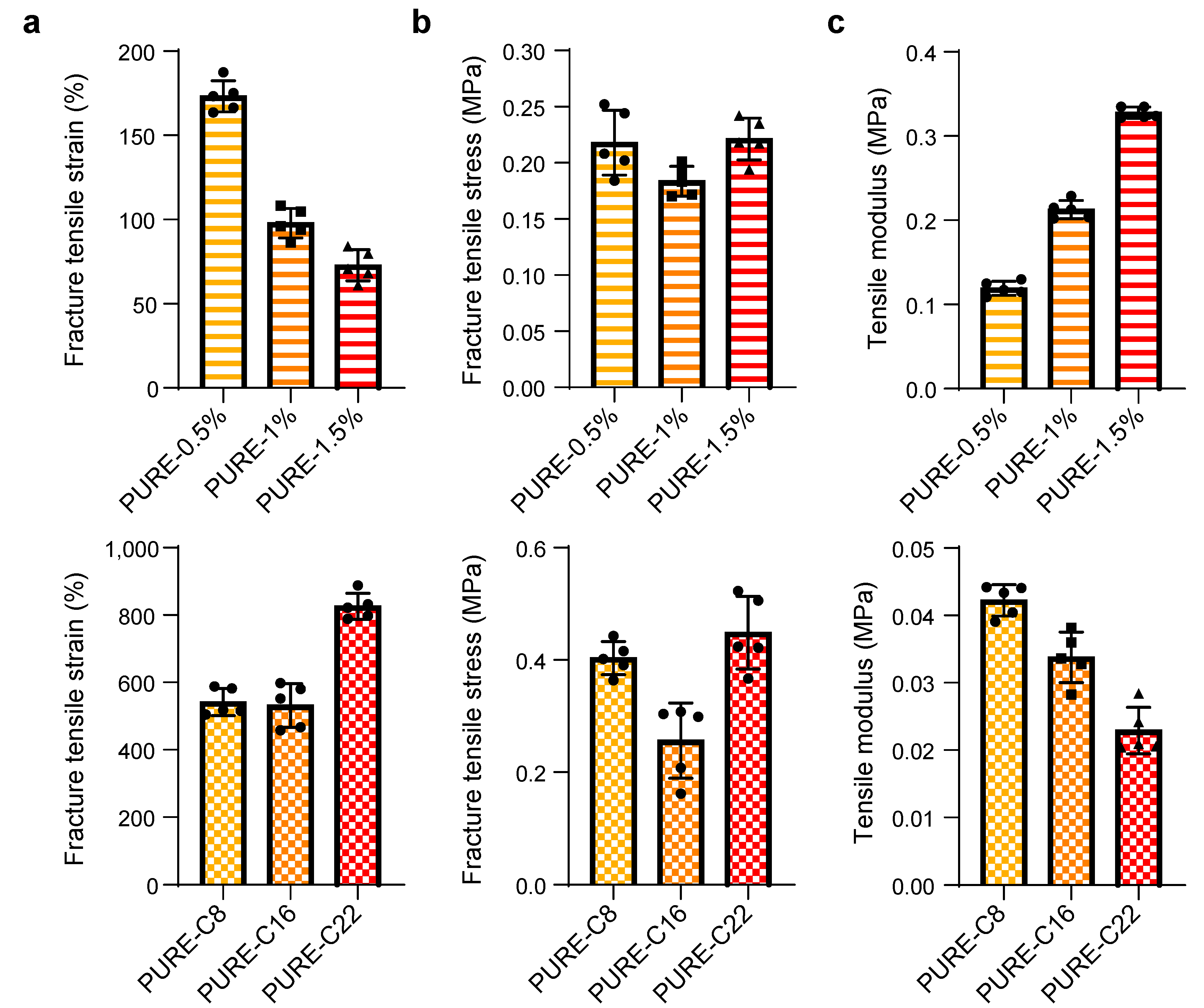


**Supplementary Fig. 8 | Characterization of mechanical properties of PURE elastomers at room temperature (25°C). Fracture tensile strain (a), fracture tensile stress (b), and tensile modulus (c) of PURE elastomers.** Bars indicate mean ± s.d. *n* = 5 elastomers per group.


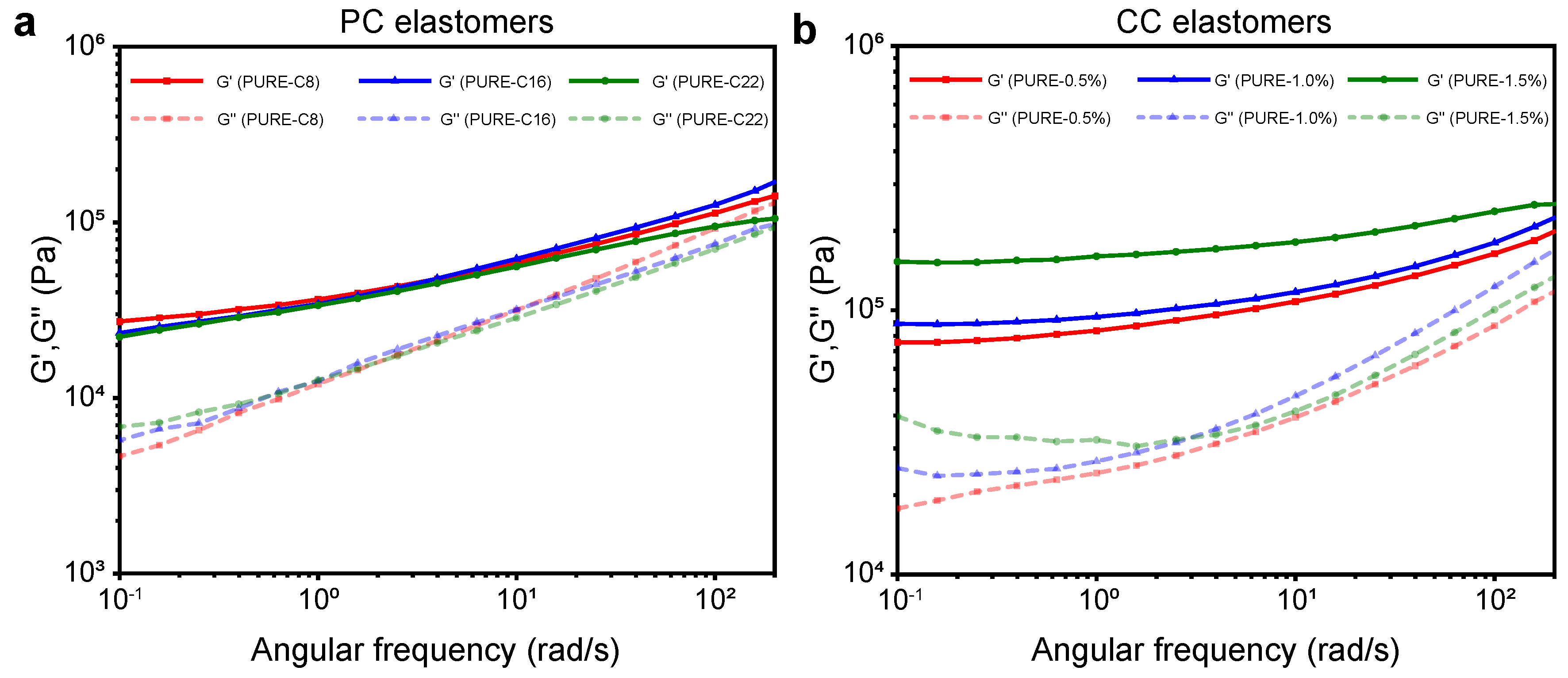


**Supplementary Fig. 9 | Rheological frequency–sweep curves of PC (a) and CC (b) elastomers at 25°C.** The G′ and G′′ relationship at 0.1 Hz was used to determine the rheological state of the sample at the experimental quasi-static timescale.


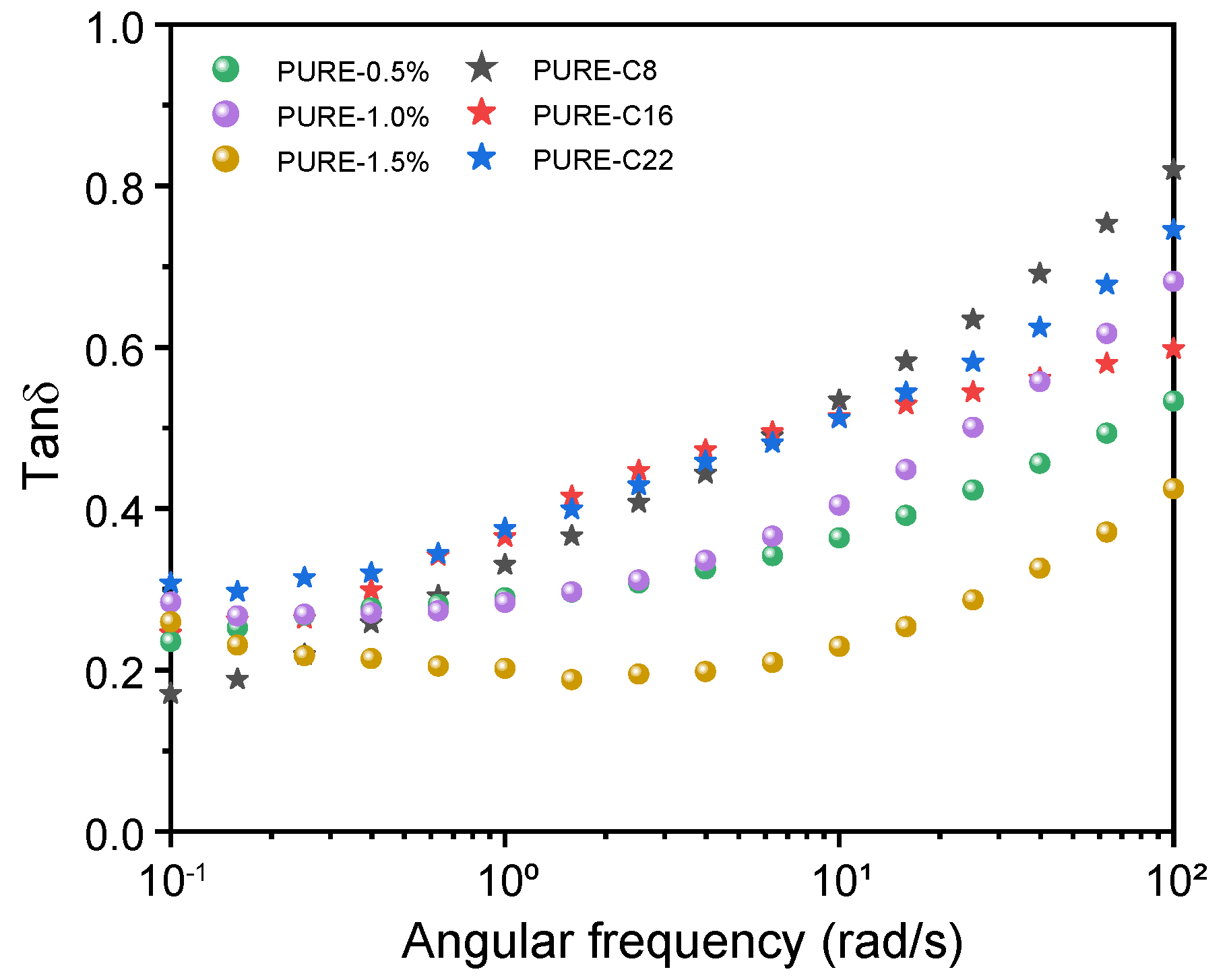


**Supplementary Fig. 10 | The loss factor measured from rheological frequency–sweep curves of PURE elastomers at 25°C.** The tan δ values at 1 Hz were employed to evaluate the damping performance at the typical human motion frequency.


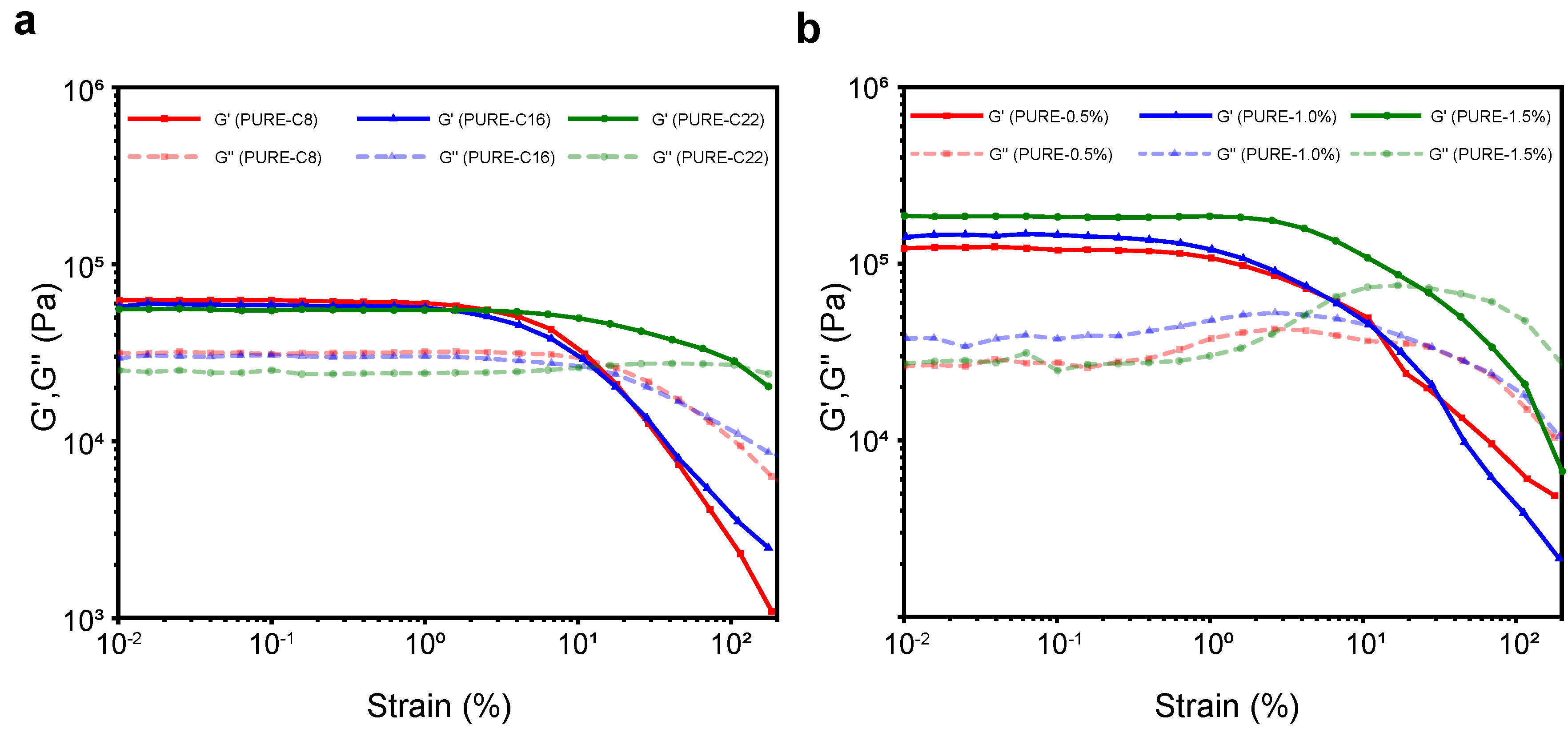


**Supplementary Fig. 11 | Rheological strain amplitude sweep curves of PC (a) and CC (b) elastomers at 25°C.**


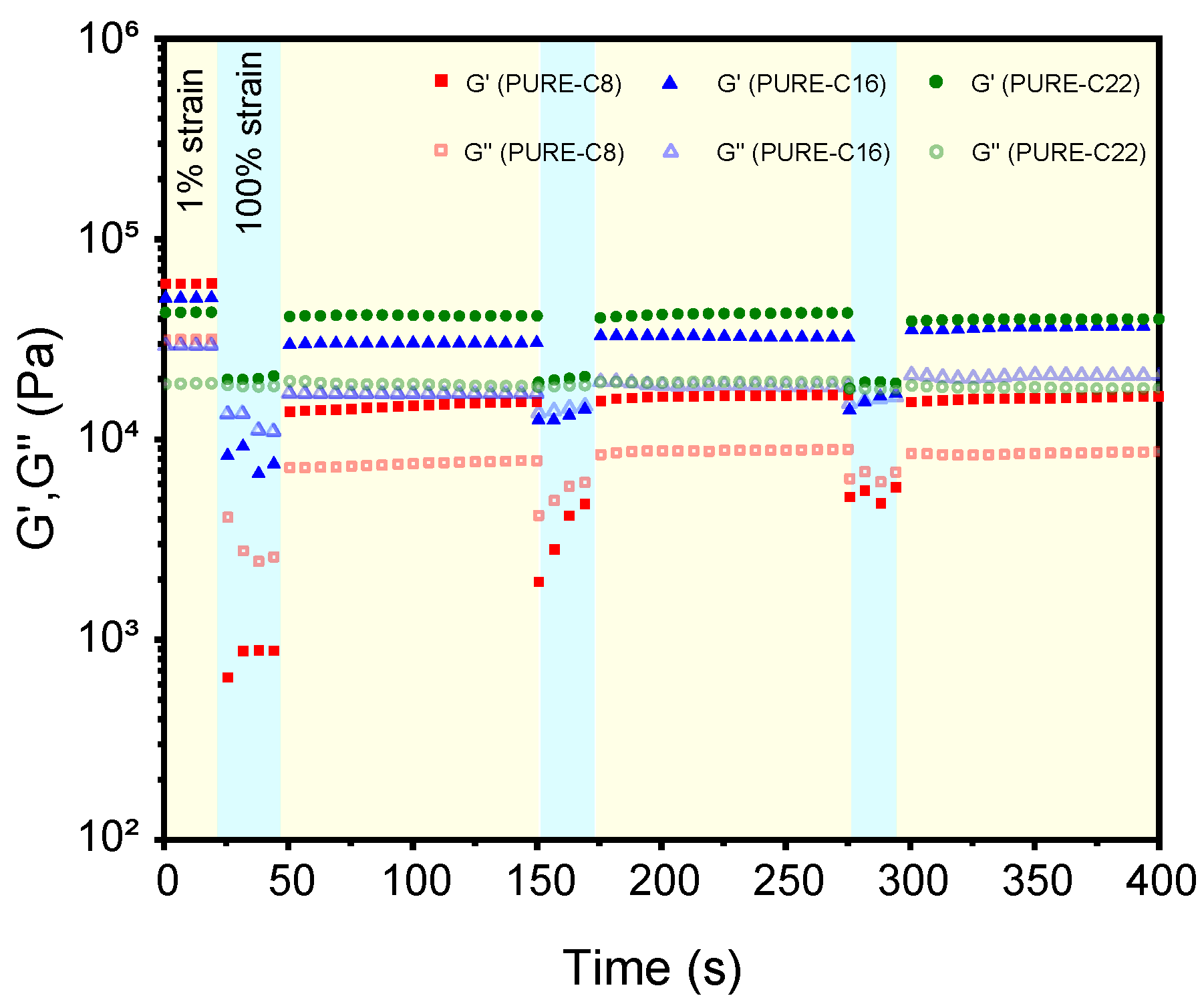


**Supplementary Fig. 12 | Cyclic strain step sweep curves of PURE elastomers at 25°C.**


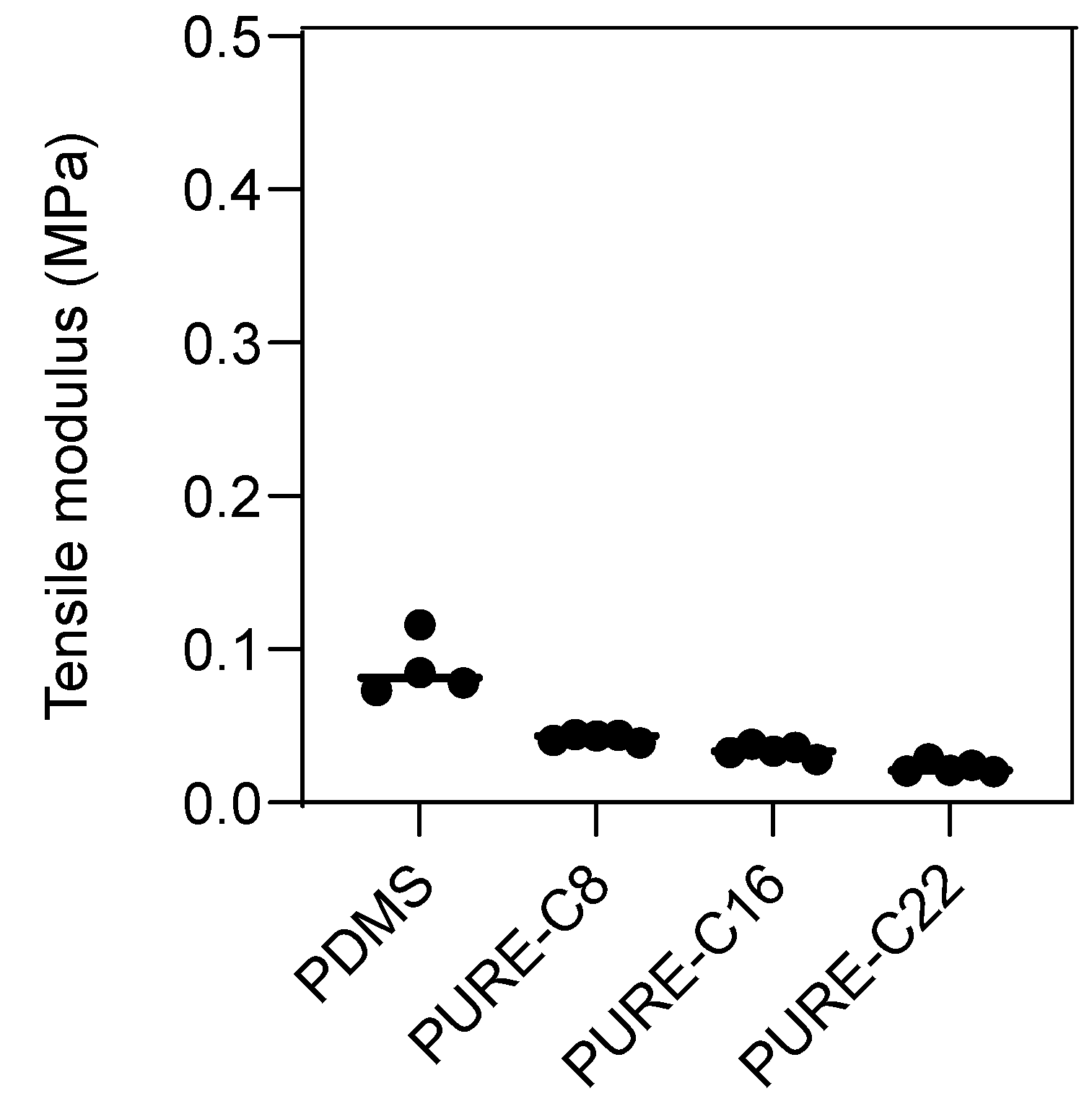


**Supplementary Fig. 13 | The adjusted modulus of PDMS.** Bars indicate mean ± s.d. *n* = 4-5 elastomers per group.


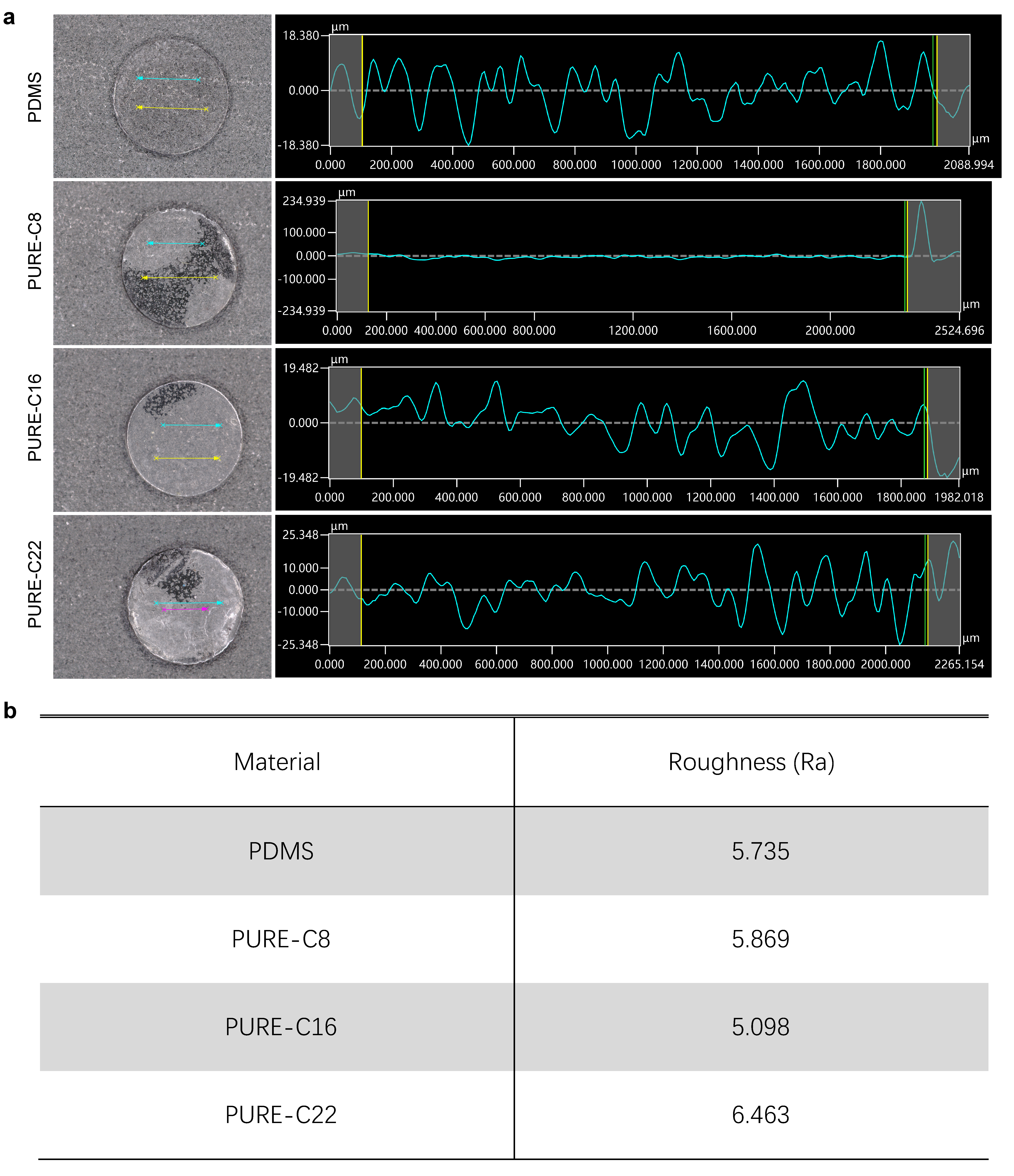


**Supplementary Fig. 14 | The non-contact profilometry analysis of PURE elastomers and PDMS discs. a.** Surface roughness analysis. **b.** The quantification for implant surface roughness.


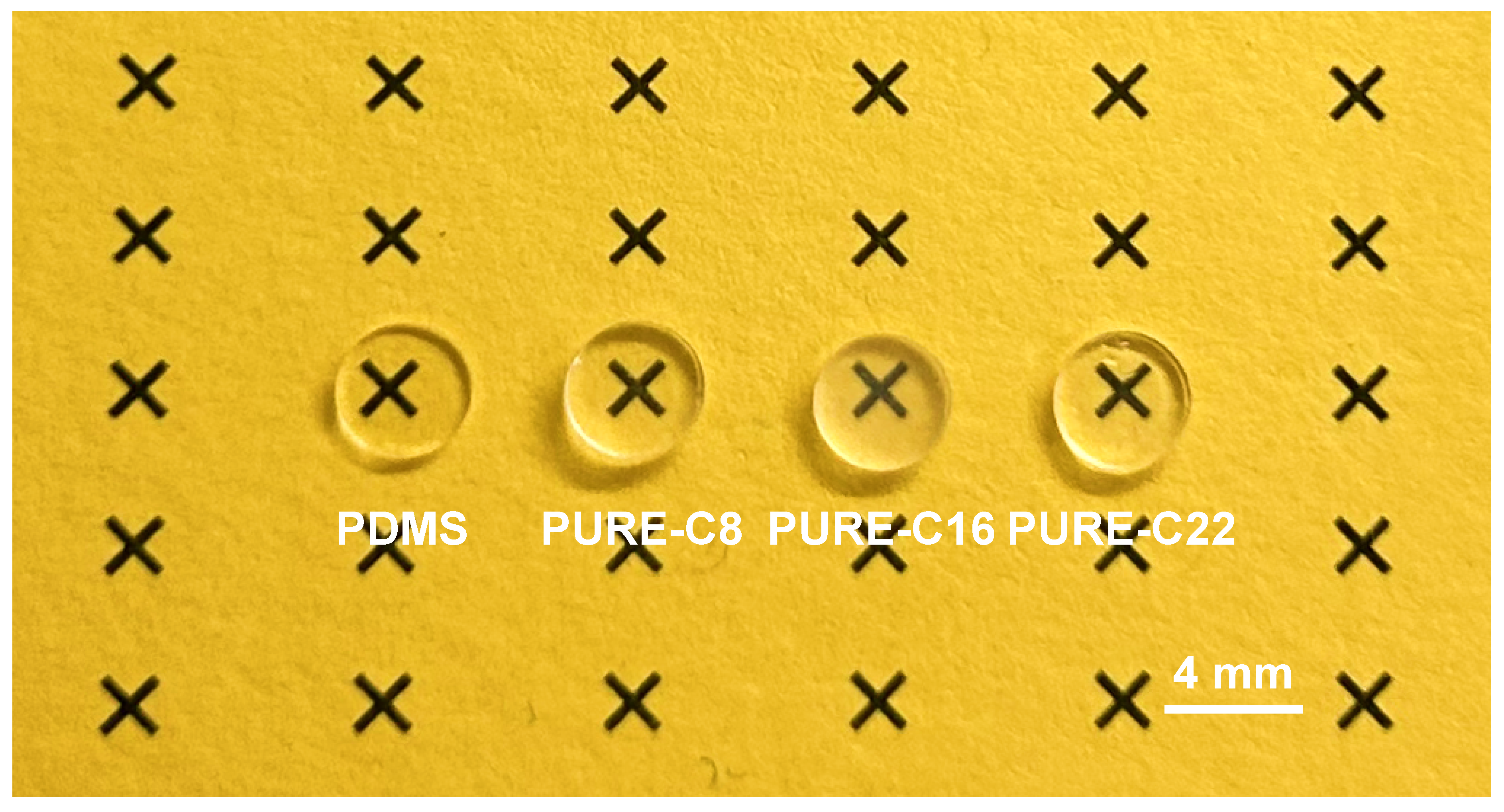


**Supplementary Fig. 15 | Appearance and size of PDMS and PURE elastomers discs for subsequent animal experiments.** All implants have a thickness of 1 mm.


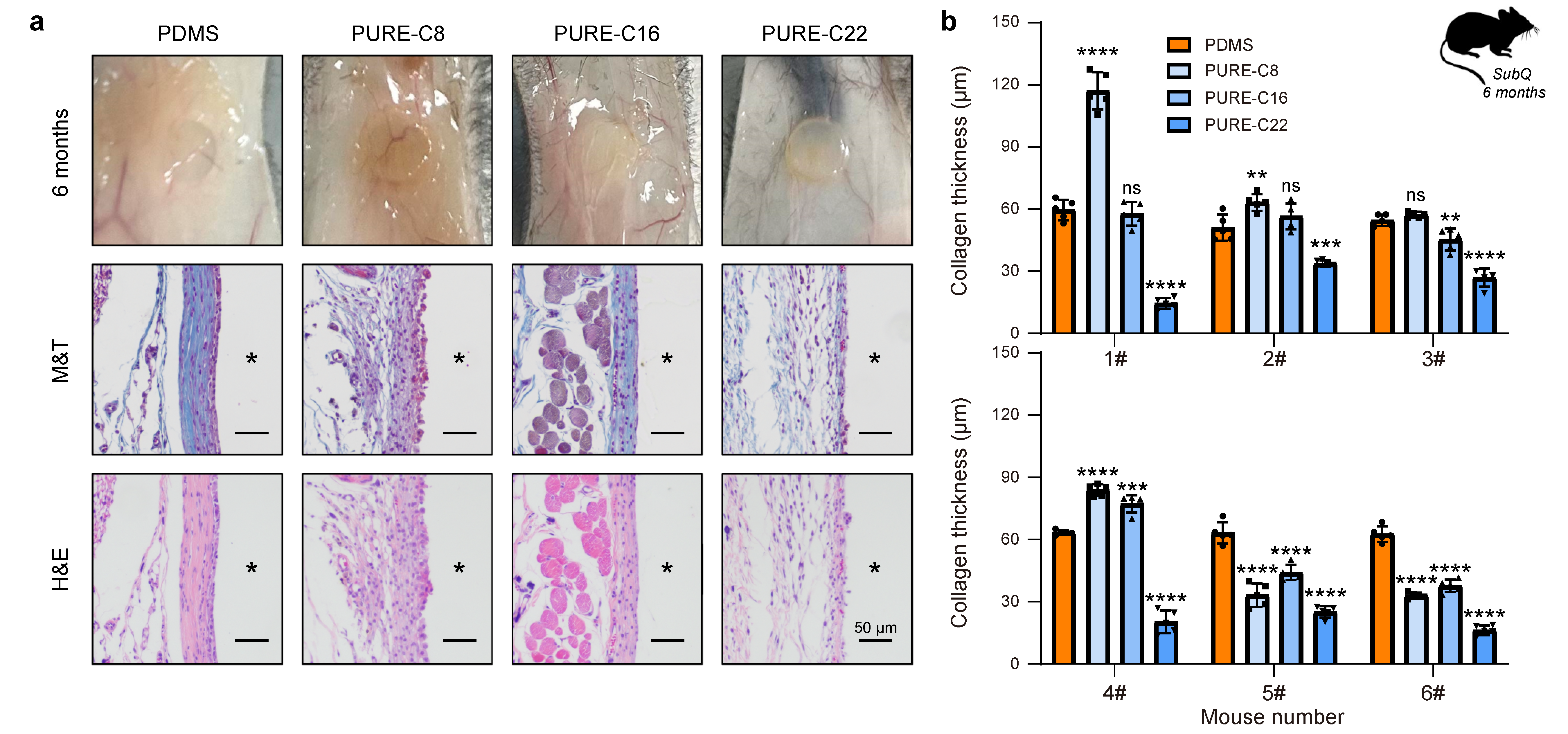


**Supplementary Fig. 16 | a. H&E and Masson’s trichrome-stained (M&T) histologic tissue sections surrounding subcutaneous elastomer implants that were explanted after 6 months in C57BL/6 mice.** The asterisk sign "*" in the section images denotes the original locations of the implants. b. Thickness of the collagen capsule in each mouse (*n* = 6 biologically independent replicates, mean ± s.d.).

**Supplementary Tables:**

**Supplementary Table 1. The components used in the preparation of the PURE elastomer.**

| PURE elastomers | Components | | | |
| --- | --- | --- | --- | --- |
|  | HPEA monomer weight (mg) | Alkyl acrylate weight (mg) | Chemical crosslinker weight (mg) | Photoinitiator weight (mg) |
| PURE-C8 | 1315 | 135 | 0 | 7.25 |
| PURE-C16 | 1245 | 205 | 0 | 7.25 |
| PURE-C22 | 1200 | 250 | 0 | 7.25 |
| For PC elastomers, the feed ratio of HPEA is 90 mol% and the feed ratio of alkyl acrylate is 10 mol% | | | | |
| PURE-0.5% | 1450 | 0 | 7.25 | 7.25 |
| PURE-1% | 1450 | 0 | 14.5 | 7.25 |
| PURE-1.5% | 1450 | 0 | 21.75 | 7.25 |

**Supplementary Table 2. Summary of the mechanical properties of PC and CC elastomers (measured at a stretching speed of 10 mm min^-1^).**

| PURE  elastomers | Tensile modulus (MPa) | Fracture tensile strain (%) | Fracture tensile stress (Mpa) |
| --- | --- | --- | --- |
| PURE-C8 | 0.042±0.002 | 541.6±35.8 | 0.403±0.026 |
| PURE-C16 | 0.034±0.003 | 531.2±57.8 | 0.256±0.060 |
| PURE-C22 | 0.023±0.003 | 825.8±34.8 | 0.448±0.058 |
| PURE-0.5% | 0.119±0.007 | 173.2±8.3 | 0.218±0.026 |
| PURE-1% | 0.213±0.010 | 97.9±7.8 | 0.184±0.012 |
| PURE-1.5% | 0.328±0.006 | 72.8±8.2 | 0.221±0.017 |

**Supplementary Table 3. Summary of the dissipation energy and dissipation ratios of PC elastomers (measured at a stretching speed of 10 mm min^-1^).**

|  | | Dissipation ratio (%) | | Dissipation energy (kJ/m^3^) | |
| --- | --- | --- | --- | --- | --- |
| PURE-C8 | 17.1 | | 3.91 | |  |
| PURE-C16 | 15.3 | | 2.37 | |  |
| PURE-C22 | 13.8 | | 2.83 | |  |

**Supplementary Table 4. The monomer feed ratio in the polymerization kinetics experiment.**

| Reaction time/h | Monomer feeding weight/mg  (HPEA+ C8/C16/C22) | Initiator weight/mg |
| --- | --- | --- |
| 0-2 (30% conversion target) | (906+94)/(861+139)/(825+175) | 5 |
| 2-8 (60% conversion target) | (906+94)/(861+139)/(825+175) | 5 |
| 8-24 (90% conversion target) | (906+94)/(861+139)/(825+175) | 5 |
